# Strawberry atlas: *Fragaria vesca* gene expression atlas for strawberry genomics

**DOI:** 10.7717/peerj.20740

**Published:** 2026-02-05

**Authors:** Minto Odagiri, Chonprakun Thagun, Takeshi Kurokura, Tomohiro Suzuki, Yutaka Kodama, Yoshinori Fukasawa

**Affiliations:** 1 Center for Bioscience Research and Education, Utsunomiya University, Utsunomiya, Japan; 2 School of Agriculture, Utsunomiya University, Utsunomiya, Japan; 3 Graduate School of Regional Development and Creativity, Utsunomiya University, Utsunomiya, Japan

**Keywords:** *Fragaria vesca*, Wild strawberry, Expression atlas, Structural variants, Housekeeping gene

## Abstract

**Background:**

*Fragaria vesca* (woodland strawberry) is a diploid model for cultivated strawberry (*Fragaria* × *ananassa* Duch. ex Rozier). While high-quality genome assemblies exist for key accessions such as Hawaii-4 (H4) and YW5AF7/Yellow Wonder (YW), a comprehensive understanding of transcriptional regulation across tissues and genotypes has been limited. Previous expression resources focused on select organs or individual studies, limiting tissue-level comparisons. Moreover, structural variants (SVs) and transposable elements (TEs), known to shape gene expression in other plants, remain understudied in *F. vesca*. An integrated gene expression resource spanning multiple tissues is needed to address these gaps and support functional genomics in strawberry research.

**Methods:**

After quality control that removed mutant and infected samples, 233 high-quality RNA-seq libraries covering more than 50 tissues and developmental stages were retained. These libraries were consolidated into nine tissue categories: leaf, root, anther, carpel, flower bud, seed, stem, early fruit, and late fruit (mature fruit). In-house leaf libraries were used as a reference to validate data structure and batch correction. Transcript abundance was quantified against the latest *F. vesca* reference genome (v6), and dimensionality reduction *via* UMAP was used to assess tissue clustering. Tissue-specific (TS) and housekeeping (HK) genes were identified based on fold-change, adjusted *p*-values, and the tau tissue specificity index. To evaluate the relationship between genome structure and expression, SVs were detected between H4 and YW using assembly- and mapping-based approaches. Genes overlapping SVs were assessed for expression trends. A web interface was developed to facilitate interactive exploration.

**Results:**

The resulting expression atlas captures a certain level of expression for ∼99% of 36,173 annotated genes that include TS genes. It also reveals robust clustering by tissue, underscoring the biological coherence of the integrated dataset. Expression profiling identified tissue-enriched genes in photosynthesis, flavonoid biosynthesis, and fruit ripening, and a core set of 719 stably expressed HK genes. SVs were significantly underrepresented in coding exons, and genes overlapping SVs had reduced expression. One example, *FvesChr6G00002800* (*FvH4_6g02210*), exhibited exonization of a TE-derived region in H4, supported by multiple datasets. The expression of GA20-oxidase family members revealed functional partitioning: *GA20ox3* was seed-specific, *GA20ox2* was active in early fruit, and *GA20ox1* showed broad, low-level expression across tissues, consistent with a subfunctionalization model. To support knowledge integration, we provide a v4-to-v6 gene ID conversion tool. All data, including SV and TE annotations, are available *via* a public web portal: https://strawberryatlas.org/easy_gdb, enabling rapid cross-tissue expression analysis.

## Introduction

*Fragaria vesca*, commonly known as woodland strawberry, is a diploid wild strawberry species widely used as a genetic and genomic model for the cultivated octoploid strawberry, *Fragaria* × *ananassa* Duch. ex Rozier (Alger et al., 2021). A high-quality reference genome and annotation for *F. vesca* (accession ‘Hawaii-4’; H4) was first published in 2011 and improved to telomere-to-telomere quality recently (Shulaev et al., 2010; Edger et al., 2018; Li et al., 2019; Zhou et al., 2023). Nonetheless, relying on a single reference genotype fails to capture the rich genetic and phenotypic diversity present in *F. vesca* (Alger et al., 2021). Different *F. vesca* accessions exhibit considerable variation in important traits such as fruit color, flowering habit, and runner (stolon) production (Shulaev et al., 2010; Tennessen et al., 2014). For example, the commonly studied H4 produces cream-colored fruit and numerous stolons (runners), whereas the popular model accession YW5AF7/Yellow Wonder (YW) also bears cream-colored fruit but is runnerless (Slovin, Schmitt & Folta, 2009; Joldersma et al., 2022). Such phenotypic traits are governed by genetic differences (Hawkins et al., 2016; Tenreira et al., 2017).

Despite the availability of reference genomes for H4 and other accessions (Alger et al., 2021; Joldersma et al., 2022; Zhou et al., 2023) and previous efforts to investigate woodland strawberry transcriptomes, our understanding of gene expression patterns in *F. vesca* remains incomplete. Importantly, the community is now adopting the latest telomere-to-telomere H4 (v6) reference (Zhou et al., 2023) for downstream expression analyses; for example, a recent AP2/ERF gene-family study was conducted on v6 (Wei et al., 2024). This shift creates a practical need for gene-ID harmonization between v6 and v4 so that legacy resources and new T2T-based studies can be integrated. Gene expression atlases, which are essential for understanding the transcriptome, have been developed for numerous species (Kapushesky et al., 2010; Sato et al., 2011; Papatheodorou et al., 2020). In *F. vesca*, eFP browser mainly focusing on flower and fruit represents a pioneering effort that provides a valuable baseline (Hawkins et al., 2017); however, it focused on specific datasets, leaving many newly available RNA-seq datasets underutilized. Currently, researchers must individually conduct cross-tissue analyses involving roots and other vegetative organs. Moreover, although transcriptome-focused initiatives for Rosaceae fruits have been recently developed (Li, Mount & Liu, 2023), comprehensive multi-tissue resources for *F. vesca* that consolidate multiple studies have been limited; here we assemble a consolidated atlas aligned to the latest T2T genome (v6) and harmonized to v4.

Another layer of complexity in gene regulation comes from transposable elements (TEs) and structural variants (SVs) in the genome. TEs are mobile sequences that can insert into new locations, sometimes disrupting genes or altering their expression (Yu et al., 2024). Similarly, SVs can rearrange the genome architecture and potentially influence gene function (Alonge et al., 2020; AlAbdi et al., 2023). In plants, TEs have been shown to impact phenotype and gene expression significantly; for example, a long terminal repeat (LTR)-retrotransposon insertion upstream of a fruit color regulator (*MdMYB1*) in *Malus domestica* (apple) triggers ectopic expression, turning golden apples red (Zhang et al., 2019). TEs can also modify gene expression by affecting splicing (Huang et al., 2015). Given that *F. vesca* harbors repetitive elements and shows intraspecific genome variation (Hawkins et al., 2016), it is pertinent to ask how these genomic changes correlate with gene expression. Understanding whether and how such TEs and SVs contribute to regulatory variation remains an important open question for the strawberry research community.

*F. vesca* is considered one of the progenitors of the cultivated strawberry, and elucidating its gene expression and regulatory mechanisms can inform key genetic pathways for crop improvement. In this study, we curated over 500 RNA-seq datasets from public repositories, supplemented with newly obtained H4 leaf samples. This curation process allowed us to construct a high-quality dataset that enabled a robust study of gene expression and its relationship to structural variation within the genome. Consequently, our resource facilitates cross-tissue and cross-genotype studies in *F. vesca* from a gene expression-focused perspective.

## Materials & Methods

### Plant materials and RNA sequencing

The Hawaii-4 (H4) accession of *Fragaria vesca* was grown under controlled conditions in a growth chamber. Fully expanded and unfolded leaves were harvested from healthy plants in biological triplicates. Tissues were flash-frozen in liquid nitrogen and total RNA was extracted using the Maxwell RSC Plant RNA Kit following the manufacturer’s protocol, including the recommended DNase treatment to eliminate genomic DNA. For each sample, Poly(A)+ mRNA was enriched and fragmented, and cDNA libraries were constructed following Illumina TruSeq protocols (including adapter ligation and PCR amplification). Libraries were sequenced on an Illumina NovaSeqX platform to generate 151 bp paired-end reads. NGS library preparation and sequencing were carried out by Macrogen Inc. (Tokyo, Japan).

### qPCR validation of tissue-biased expression

Total RNA was extracted from each tissue using the Maxwell RSC Plant RNA kit together with the Maxwell RSC instrument (Promega, Madison, WI, USA) according to the manufacturer’s instructions. Extracted RNA was quantified using a NanoDrop spectrophotometer (Thermo Scientific, Waltham, MA, USA), and 500 ng of RNA was reverse-transcribed into cDNA with ReverTra Ace (Toyobo, Tokyo, Japan) according to the manufacturer’s manual. Real-time qPCR was performed on a qTOWER^3^ 84 system (Analytik Jena, Germany) using the KAPA SYBR Fast qPCR kit (KAPA biosystems, USA). Primer sequences used for qPCR were as follows: *FvTFL1* (Fw, 5′-CTGGCACCACAGATGCTACA-3′; Rv, 5′-AACGGCAGCAACAGGAAC-3′) (Koskela et al., 2012).

### Public dataset collection for multi-tissue atlas

To build a multi-tissue expression profile of *F. vesca*, we retrieved publicly available RNA-seq datasets from NCBI Sequence Read Archive (SRA) in March 2024. We included data from multiple tissues and developmental stages, focusing on those generated from *F. vesca*. Samples that had undocumented tissue types (*i.e.,* the tissue attribute was missing in the sample metadata) or employed library strategies other than RNA-seq (*e.g.*, whole genome sequencing) were omitted. To obtain expression data with minimal bias, we selected samples based on metadata from publicly available datasets, excluding mutant and experimentally infected samples and retaining only those derived from wild-type or control conditions. Initial screening was conducted using AMALGKIT ver. 0.11.3 (Fukushima & Pollock, 2020). To better understand clustering by tissue or stage, we performed a dimensionality reduction with UMAP (McInnes, Healy & Melville, 2018) on the expression dataset. UMAP allowed us to see if the samples grouped primarily by tissue type. We expected that biological tissue type would be the dominant factor, given that different tissues have very distinct transcriptomes. Tissues with low sample counts were merged into closely related major tissue categories after confirming similar expression profiles (*e.g.*, crown was merged into stem after UMAP showed high similarity, Fig. S1). We assessed cross-study batch effects and outliers. Outliers were removed by the AMALGKIT quality-control filters (Fukushima & Pollock, 2020), and cross-study effects were controlled by applying surrogate variable analysis (SVA) to the log_2_-TPM atlas matrix (AMALGKIT ver. 0.11.3, using the default SVA settings), because no negative-control (*e.g.*, housekeeping) genes were available. Following AMALGKIT outlier filtering, tissue categories that were left with too few residual samples were not retained; the remaining samples in those categories were excluded from downstream atlas analyses. For robustness, we also generated a count-based representation by summarizing pseudo-alignment output to tximport-derived gene counts and applying the DESeq2 variance-stabilizing transformation (VST). This analysis yielded the consistent tissue-level structure (Fig. S2). The complete list of public datasets and detailed information are summarized in Table S1. After assembly of this compendium, we had an expression matrix covering multiple tissue types and, indirectly, multiple genotypes.

### Identification of SVs and TEs

To investigate the influence of genomic variations, we identified SVs and TEs in the *F. vesca* genome, focusing on their overlap with gene regions. We used the *F. vesca* reference genome sequence and gene annotation obtained from the Genome Database for Rosaceae (GDR) (Jung et al., 2019).

**SV detection:** We primarily identified SVs between the H4 and YW genomes by leveraging the newly available YW genome assembly (Joldersma et al., 2022) and telomere-to-telomere H4 genome (Zhou et al., 2023). Given the higher expected accuracy of assembly-based approaches (Liu et al., 2024), we used SVIM-asm as our primary method for SV detection (Heller & Vingron, 2021). Specifically, the YW genome assembly was aligned to the H4 reference genome using minimap2 ver. 2.28-r1209 (Li, 2018) with recommended parameters (-x asm5) and subsequently scanned the alignment for SVs using SVIM-asm. To build a high-confidence SV catalog for YW, we retained only SVIM-asm variants that achieved cross-platform confirmation, *i.e.,* were independently supported by mapping-based callers on each platform: Manta v1.0.3 on Illumina whole-genome reads (SRR26113965; generated in a different project but derived from the same inbred line, YW) and Sniffles2 v2.0.7 on Oxford Nanopore reads (SRR21142895; same biological sample as the YW assembly). Variants detected by all three callers (SVIM-asm, Manta, and Sniffles) were retained and classified as tier 1. Because insertion detection for variants over 500 bp is challenging with short-read-based methods (Delage, Thevenon & Lemaitre, 2020), SVs in this size range supported by both SVIM-asm and Sniffles were retained separately as tier 2. Together, tier 1 and tier 2 comprise 776 high-confidence SVs out of 2,339 SVIM-asm calls, which were used for downstream analyses. The remaining SVs, categorized as tier 3, were not used for further analysis but are included in the dataset for completeness and availability. SV calls were selected and merged using SURVIVOR ver. 1.0.7 (max_distance = 1 kb, same_type = 1).

**TE annotation:** We employed *de novo* approaches to annotate TEs in the latest *F. vesca* genome (Zhou et al., 2023). TE library computed *de novo* by RepeatModeler ver. 2.0.4 was clustered at 80% to consolidate subfamily into family level groups using cd-hit ver. 4.8.1 (Li & Godzik, 2006; Goubert et al., 2022). This 80% threshold follows the widely used “80-80-80” rule, providing a practical balance between removing redundancy and avoiding over-splitting (Goubert et al., 2022). For each family, a representative sequence was classified into major TE categories (class/order/superfamily) using RepeatModeler. Sequences remaining ambiguous at the class level were further assigned with DeepTE (Yan, Bombarely & Li, 2020), following a recently adopted procedure (Brown et al., 2025). Repeat annotation was then conducted using RepeatMasker ver. 4.1.5 (http://www.repeatmasker.org) with the clustered TE library.

### Classification of SV

Given the broad and complex range of SV lengths, we further classified them based on TE content using a simple statistical model to understand if certain size classes correlate with TE-mediated events. Using a normal approximation to the binomial distribution, we evaluated the probability that a given genomic region contains a TE proportion greater or less than expectation. The TE proportion in a randomly sampled genomic region of length *l* follows approximately *N(p, p(1-p)/l)*, where *p* is the genome-wide background TE proportion. Regions exhibiting TE proportions significantly deviating from *p* were identified using a standard normal test, with the upper tail probability indicating TE-derived regions and the lower tail probability indicating TE-depleted regions. In regions with a significantly high abundance of TEs (*i.e.,* TE-derived regions), those in which a single TE family accounted for over 80% were classified as occupancy-type, while the remaining regions were designated as TE-complex.

We defined an SV as harbored by a gene if any portion of the SV overlapped that gene’s annotated region or fell within 2 kb upstream from those annotated region (putative promoter).

### Statistical analysis of SV and TE distribution

To determine if SVs show a non-random distribution with respect to gene regions across the genome, we carried out statistical tests as follows. We first calculated the proportion of the genome occupied by exonic, intronic, and intergenic sequences using the genome annotation. For each SV annotation, we then determined whether it overlaps gene features using the genome annotation general feature format (GFF) file. Specifically, we intersected SV coordinates with gene exon coordinates, intron coordinates, and intergenic regions. Event-based classification (*i.e.*, count one if SV and exon/intron/intergenic are overlapped) has struggled with long SV annotations because they overlap with multiple categories (*e.g.*, exon and intron). Therefore, we simply computed the number of bases overlapping SV annotations and genic annotations. Following this criterion, we compared the observed counts to expected counts if SVs were randomly distributed according to the proportion of the genome in each category. A chi-square test for goodness-of-fit was used to assess whether the deviations between observed and expected counts were significant. For TEs, the same approach was taken to test a non-random distribution of TE with respect to coding regions. All analyses were conducted in the R environment and with standard libraries for statistical computing.

### Read mapping and expression quantification

All RNA-seq reads (our H4 leaf samples and the public dataset reads) were preprocessed, pseudo-aligned to the reference, and processed for outlier removal and surrogate variable analysis using AMALGKIT ver. 0.11.3 (Fukushima & Pollock, 2020), which internally employs fastp ver. 0.23.4, kallisto ver. 0.50.1 (Bray et al., 2016). We utilized the most recent telomere-to-telomere *F. vesca* reference and gene annotation (Zhou et al., 2023). Genes that failed to meet a threshold (transcripts per million (TPM) > 1) in all samples were excluded from housekeeping-gene identification analyses but were retained in the final atlas for completeness. For robustness, we also prepared a count-based representation by summarizing pseudo-alignment output to tximport-derived gene counts and applying DESeq2 ver. 1.42.0 VST (Love, Huber & Anders, 2014); this produced the consistent tissue-level structure (Fig. S2).

Paired-end YW reads (and H4 for comparisons) were additionally processed by rna-seq pipeline ver. 3.12.0 (https://nf-co.re/rnaseq/) under nf-core ver. 23.04.2 (Ewels et al., 2020) internally using STAR ver. 2.7.9a provides more tolerant alignment (Dobin et al., 2013). For the leaf-tissue analysis, we used seven H4 paired-end libraries from two independent sequencing batches (one generated in this study and one obtained from public data), along with three YW libraries from a single paired-end batch. Raw read counts were normalized for library size, and log_2_ fold change (log2FC) values were shrunken in DESeq2 ver. 1.42.0 using apeglm (Love, Huber & Anders, 2014; Zhu, Ibrahim & Love, 2019). Unwanted variations were removed using RUVSeq with housekeeping (HK) genes decided in this study (Risso et al., 2014). RUVg function and *k* = 1 was applied. We contrasted the two genotypes (H4 as baseline *vs* YW) to obtain a list of differentially expressed genes. This alignment-based analysis was restricted to paired-end libraries to leverage split-read and discordant-pair evidence near SV breakpoints; atlas-wide quantification and matrices remain kallisto-based. To verify aligner independence while keeping the differential expression (DE) model fixed, we re-ran the same DE design on a pseudo-alignment-derived gene-count matrix using tximport. The results matched the STAR-based analysis (Fig. S3).

### Identification of tissue-specific (TS) and housekeeping (HK) genes

We used the batch-corrected log_2_ transformed TPM for finding TS genes. Each of the nine major tissue categories was compared against each other to find significantly overexpressed genes using limma ver. 3.46.0 (Ritchie et al., 2015). We applied |log_2_ FC|≥ 2 and BH-adjusted *P* ≤ 0.01 to identify significantly overexpressed genes. If a gene *G* is overexpressed in a tissue *T* in comparison with the other eight tissues, *G* was considered as specifically expressed in tissue *T*. In addition, *τ* index to assess TS expression by applying a threshold of 0.85 (Lüleci & Yılmaz, 2022).

Genes that never reached TPM ≥ 1 in any sample were excluded from HK calculations (but remain in the atlas tables for completeness). For variability metrics, a gene with low expression (TPM ≤ 1) was treated as 0, following criteria used in previous studies (Hoang et al., 2017; Machado et al., 2020; Qin et al., 2024). In addition to this, to isolate genes that are robustly expressed irrespective of tissue type, we defined HK genes as those that, in every sample across all tissues, demonstrated expression levels exceeding the median of corrected log_2_ transformed TPM.

TS gene lists were first mapped to KO identifiers that had been assigned using KAAS (Moriya et al., 2007). Over-representation analysis was then carried out with clusterProfiler ver. 4.10.0 (Yu et al., 2012). KEGG pathway enrichment was performed with enrichKEGG (organism = “ko”), and KEGG module enrichment was carried out using enrichMKEGG (organism = “ko”), with both analyses using the complete pan-KEGG dataset as the background. *P*-values were corrected by the BH method, and terms with adjusted *P*-values < 0.05 were considered significant.

### Gene version mapping

To establish a robust gene map between *F. vesca* genome versions v4 and v6, we considered whole genome synteny by MCScanX and phylogenetic relationship using OrthoFinder (Wang et al., 2012; Emms & Kelly, 2019). First, we performed orthogroup inference using OrthoFinder on a dataset of representative plant proteomes, including both *F. vesca* versions and key outgroups, to place all genes within a broad phylogenetic context. Data for Rosaceae species (*Fragaria vesca* v4, v6; *Fragaria* × *ananassa*; *Rosa chinensis*; *Prunus persica*; *Malus domestica*) were obtained from GDR. Data for outgroup species (*Arabidopsis thaliana, Glycine max, and Oryza sativa*) were obtained from Phytozome (Goodstein et al., 2012). For proteomes containing multiple isoforms per gene locus, only the longest isoform was retained for all downstream analyses. The full detailed bioinformatic workflow is available at https://github.com/yfukasawa/gene_version_mapper.

## Results

### Data collection, augmentation and establishment of comprehensive gene expression atlas of *F. vesca*

A datamining process applied to public sequence repositories yielded more than 500 raw RNA sequencing datasets for *F. vesca*. Samples lacking tissue information were excluded, and to ensure that the resulting expression profiles were as representative of the wild type as possible, samples derived from mutant or infected specimens were identified and removed. In addition to the publicly available datasets, we generated in-house RNA-seq libraries from leaves collected at different developmental stages (unfolded and full expansion) under controlled conditions. These libraries were used as a reference for assessing data quality and validating batch correction procedures (see Fig. S1 and Methods). The integrated expression matrix encompassed mainly nine distinct tissue types: leaf, root, anther, carpel, flower bud, seed, stem, early-stage fruit, and late-stage fruit (equivalent to mature fruit in this study).

After filtering and correction (Fig. S5), we finally constructed an expression atlas for *F. vesca* across multiple tissues composed of 233 samples. Out of the 36,173 predicted genes in the *F. vesca* genome (Zhou et al., 2023), we found that 36,039 genes (∼99% of the annotated set) were expressed at a certain level (TPM > 1 in at least one sample) in this atlas. This indicates that the majority of annotated genes are transcriptionally active in at least one tissue, while the remaining ∼1% might represent genes with quite low expression levels, very specialized expression (in conditions not sampled), or possibly pseudogenes.

**Figure 1:**
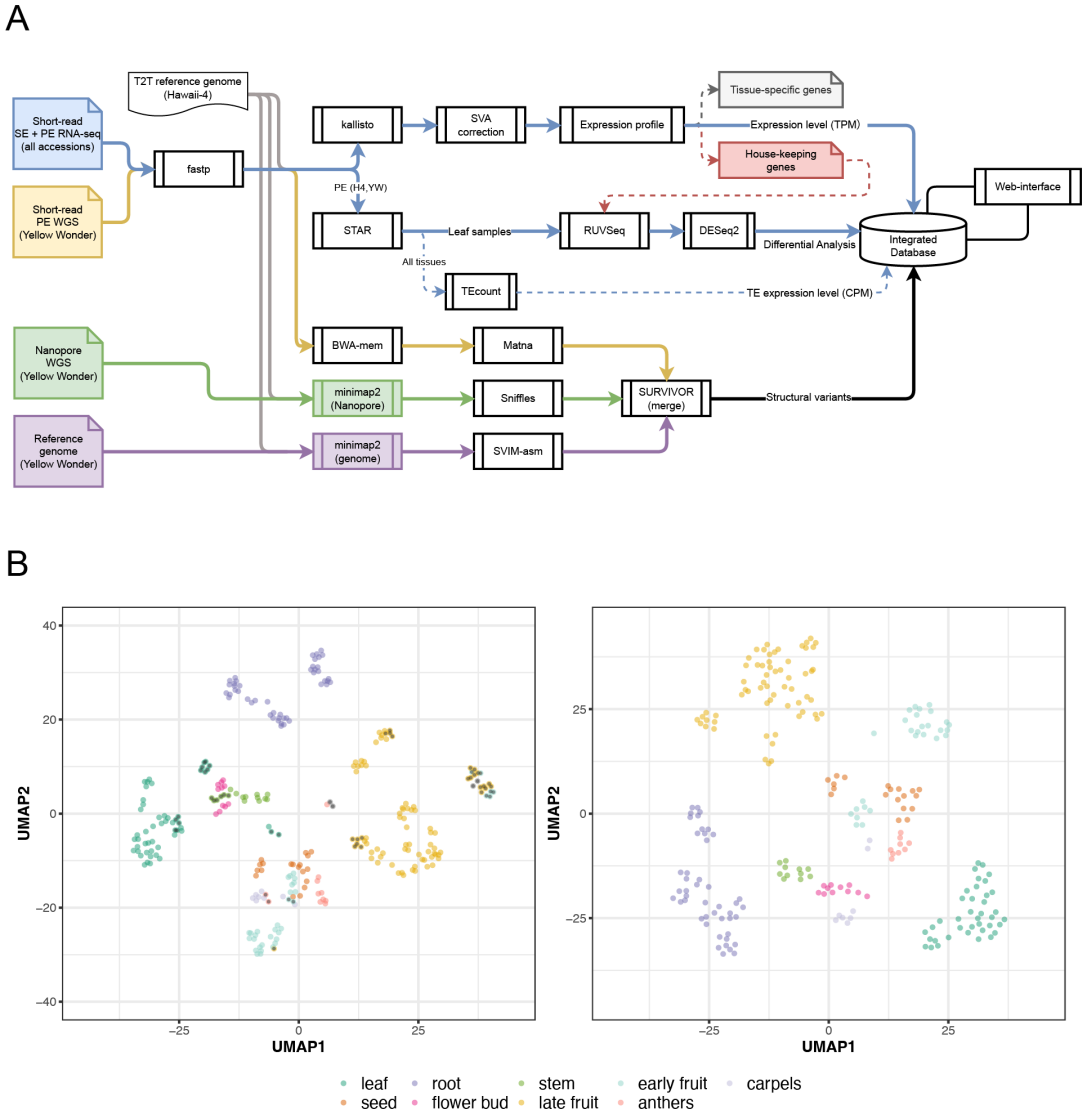
Overview of the analysis and datasets. (A) Schematic diagram illustrating the analytical workflow used to investigate wild strawberry RNA-seq and genomic datasets. (B) The left and right panels show UMAP visualization of samples before and after the batch correction, respectively. Samples with dots show those filtered.

Standard genotype information could not be obtained for 111 of the 233 samples. Among the remaining samples, however, nearly all were either H4 or YW, accessions for which reference genome sequence have been established (Joldersma et al., 2022; Zhou et al., 2023), comprising approximately 90% of the samples with available standardized genotype data (Table S1). Reads from those samples were analyzed with the reference and used for the gene expression estimation (Fig. 1A). Dimensional reduction using UMAP revealed that the expression data are primarily clustered by tissue type, underscoring that each tissue has a characteristic gene expression profile (Fig. 1B).

As an additional validation, the in-house libraries mapped unambiguously to the leaf cluster, while samples from the same developmental stage formed the tightest neighborhoods (Figs. S1–S2). Notably, even though the public libraries were generated under diverse growth conditions, the merged data still resolved primarily by developmental stage rather than by source laboratory (Figs. S1–S2). These observations confirm that the public leaf libraries are of sufficient quality and that batch correction retains true biological structure. More broadly, the full set of public samples displays biologically coherent clustering after batch correction (Fig. 1B), supporting their use in comparative expression studies. Because normalization choices can influence sample structure, we assessed robustness by comparing our TPM-based representation with a counts-based alternative (DESeq2 VST). Both approaches yielded concordant tissue-level organization: sample-sample similarity matrices were highly consistent (Fisher z-transformed Pearson and Spearman correlations ∼0.98), and hierarchical clustering produced high agreement across a broad range of cut levels (Adjusted Rand Index ∼0.95), both for biological replicates in our in-house libraries and for other public datasets (Figs. S1–S4). Taken together, within the scope of this atlas, using scaled metrics (*i.e.,* TPM) does not materially affect tissue-level conclusions. Accordingly, batch effects were removed by applying SVA to logarithm-scaled expression values.

### Tissue-specific *vs.* housekeeping genes

**Figure 2:**
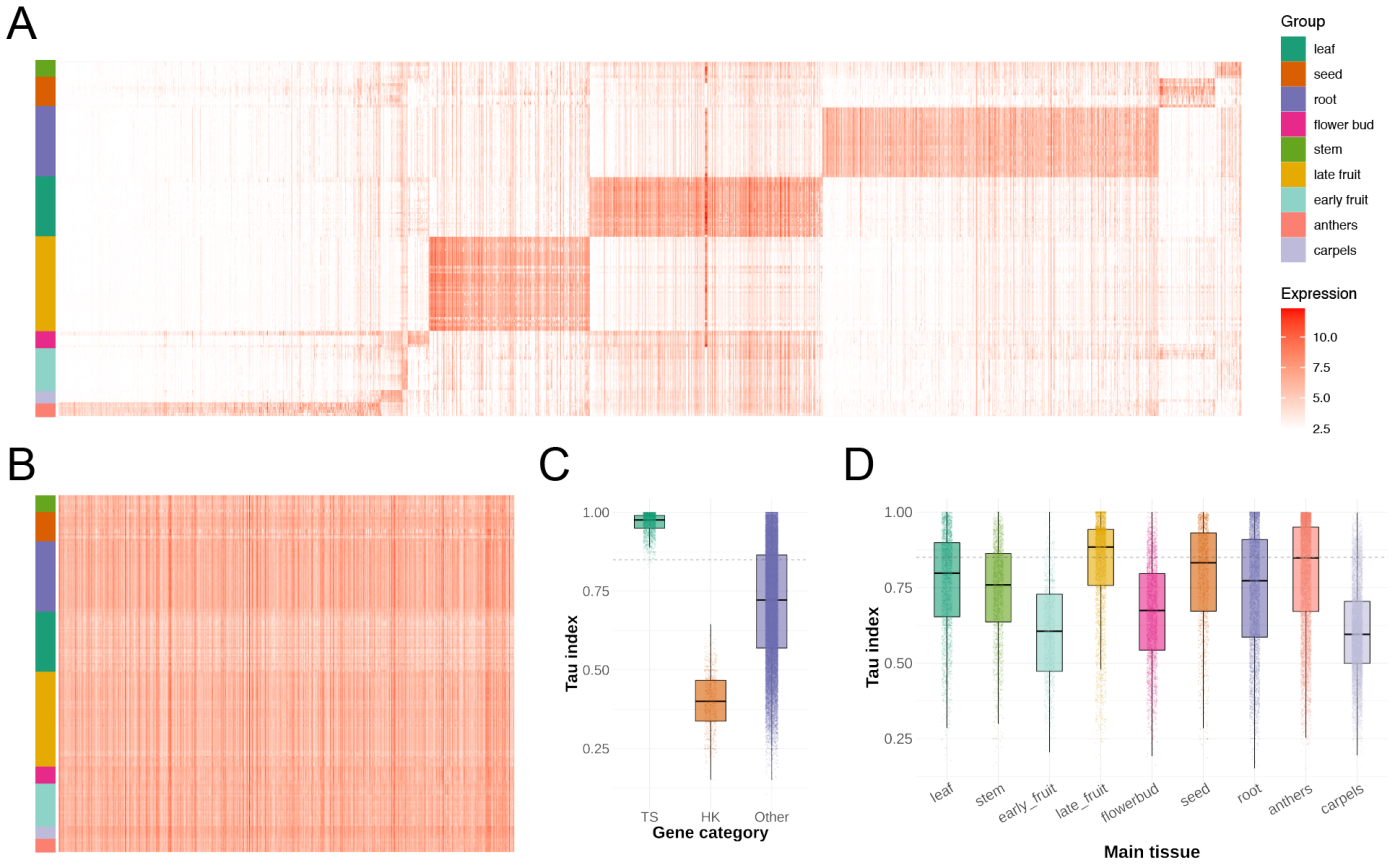
Expression patterns of TS and HK genes. (A, B) Global expression profiles of TS and HK genes, respectively. Columns and rows represent genes and samples, respectively. Expression levels are shown as log_2_-transformed TPM values. (C) Box plots illustrating the distribution of *τ* index values for TS genes, HK genes, and other genes. Data points (jittered) indicate individual gene distribution within each category. (D) Box plots illustrating the distribution of *τ* index values across each tissue type.

Within each tissue type, samples from different studies clustered together well, suggesting that tissue identity is a main determinant of expression pattern in most cases. Using a compiled expression atlas for each tissue type, TS gene identification was subsequently performed (Fig. 2A and Table S2). Genes highly expressed in leaves but low elsewhere included those involved in photosynthesis (chlorophyll a/b binding proteins, Ribulose-1,5-bisphosphate carboxylase/oxygenase (RuBisCO) subunits) and carbon fixation, whereas genes enriched in anthers, root, and stem included many related to flavonoid and phenylpropanoid biosynthesis. Similarly, in late-stage fruit tissue, the pronounced expression of genes involved in pentose and glucuronate interconversions. This demonstrates the requirements during maturation in the tissue. Pathways and modules enriched in each tissue are summarized in Table S3. Conversely, the gene expression atlas for each tissue also facilitates the identification of HK genes that are constitutively expressed across all tissues with small variation. We found that approximately 20% of genes (7,286 out of 36,173 genes) are expressed (TPM > 1) across all sampled tissues, although their expression levels can still vary. Our stability analysis across tissues identified that the most stable transcripts included ribosomal protein genes, translation factors, and components of the basal metabolism. Although typically used on smaller candidate sets, we applied them to a broad set of known and classical HK genes (*e.g.*, *actin*, *tubulin*, *ubiquitin-conjugating enzyme*, *etc.*) to see which are most stable across the multi-tissue panel. Previous studies have noted that the stability of common reference genes can vary with tissue or atypical conditions (Suzuki, Higgins & Crawford, 2000; Niu et al., 2014; Luo et al., 2024). In particular, Amil-Ruiz et al. (2013) had identified *actin* (*FaACTIN*) and *elongation factor 1-alpha* (*FaEF1α*) among the top stable genes under various conditions in *F.* × *ananassa,* arguing the expression variability of other classical HK genes (Amil-Ruiz et al., 2013). In *F. vesca*, a few genes in the classic reference gene families such as *ubiquitin-conjugating enzyme* (*UBC*) and *cyclophilin* (*CYP*) were included in the HK genes. Other classic reference gene families like *ACTIN, EF1α, tubulin* (*TUB*), *glyceraldehyde-3-phosphate dehydrogenase* (*GAPDH*), and *polyubiquitin* (*UBQ*) were indeed uniformly and highly expressed across all tissues, but most of the members in those families showed moderate expression variability. We compiled a list of 719 most stable and highly expressed genes across the multi-tissue panel (Fig. 2B and Table S4), which could serve as candidate internal controls for future gene expression studies in *F. vesca*.

Additionally, we assessed tissue specificity using the Tau (*τ*) index (Yanai et al., 2005) to determine whether the predicted HK genes exhibited broad expression patterns. The *τ* index ranges from 0 to 1, with lower values indicating ubiquitous expression and higher values reflecting tissue-restricted expression. The *τ* index for the identified HK genes fell within the range of 0.15 to 0.65, with a mean of 0.40, consistent with their stable expression across tissues. In contrast, the *τ* scores for the TS genes ranges from 0.79 to 1.0 with an average of 0.97, underscoring their TS expression pattern (Fig. 2C). The *τ* index distribution for each tissue type revealed that late-stage fruit tissue shows a higher *τ* index than the other tissues (Fig. 2D).

### Genome-wide relationship between structural variation and gene expression

**Figure 3:**
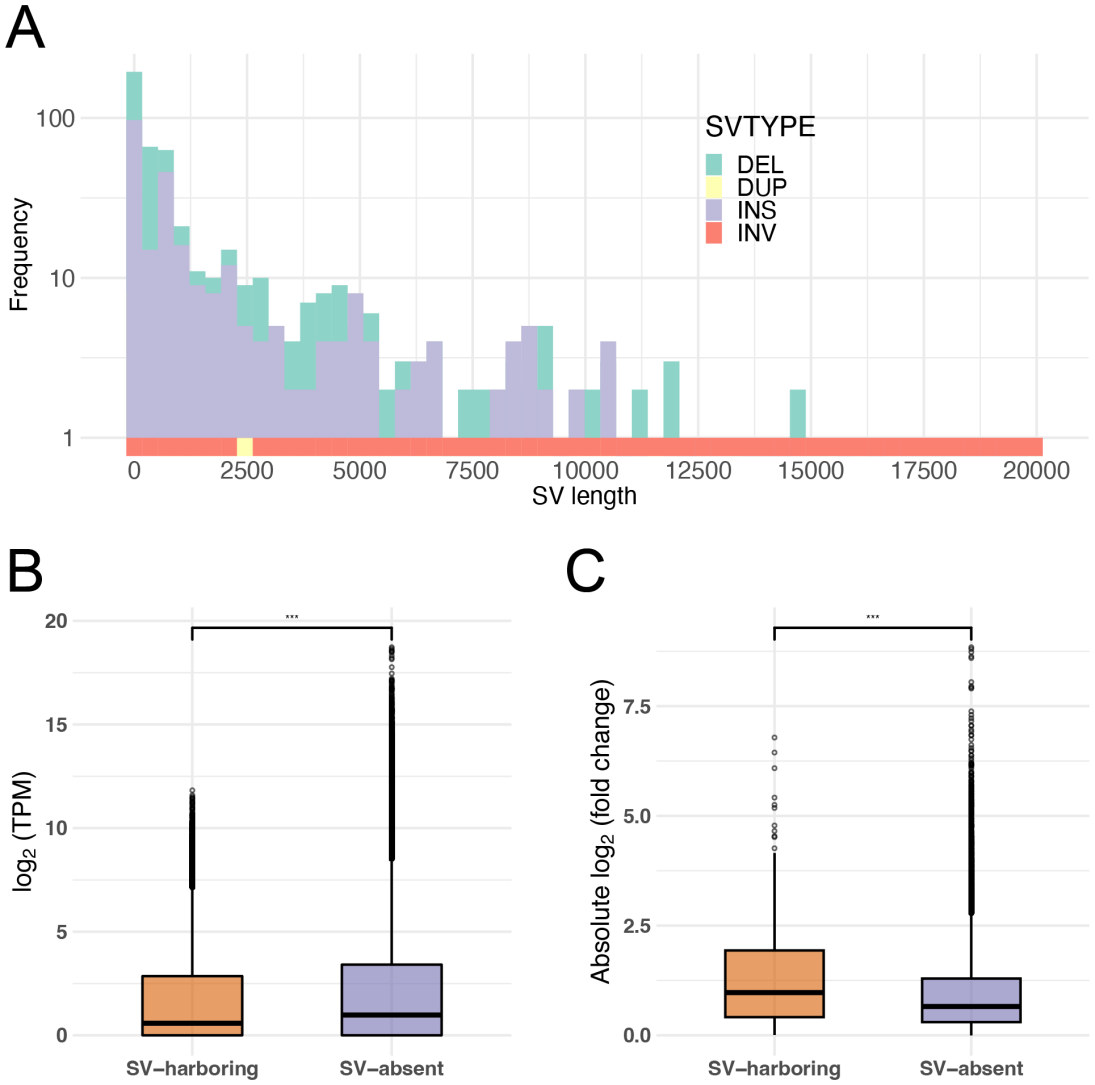
Characteristics of SVs and genes overlapping SVs. (A) Length distribution of SVs identified in this study. (B) Box plots showing the distribution of log_2_-transformed TPM in the expression atlas for genes associated or not associated with SVs. (C) Box plots showing the distribution of log_2_-transformed absolute fold-change in leaf tissue between genes associated or not associated with SVs. Significance was determined using two tailed Wilcoxon test. *P*-values are indicated by asterisks: *p* < 0.001 (***), *p* < 0.01 (**), and *p* < 0.05 (*).

The consistency of HK gene expression across studies implies that there is not widespread expression reprogramming with different genetic backgrounds, at least for HK genes (Fig. 2B). The compiled dataset, which included experiments from different labs and in some cases different *F. vesca* accessions, also allowed a preliminary look at expression variation attributable to genotype or environment. While point mutations or small indels in regulatory regions can explain modest expression shifts, significant changes often arise from larger-scale genomic variations in other plant species (Alonge et al., 2020). To explore this possibility, we aligned each accession’s genome assembly to investigate SVs across the genome of *F. vesca*. A reliable set of SVs, including insertions, deletions, and a small number of duplications, was identified between the H4 and YW genomes. The SVs ranged from 50 bp to approximately 20 kbp. A gradually decaying distribution was observed with peaks identified around 2 kbp and 5 kbp in length (Fig. 3A). When examining these SVs relative to genes, we first confirmed an underrepresentation from exons (Table S5). Quantitatively, the percentage of SVs overlapping exons was significantly lower than expected by chance, reinforcing the idea that exons are protected from large genomic alterations in *F. vesca* genome (chi-square test, *p*-value < 2.2e−16). We also looked at how many genes have any SV in or near them. In fact, a very minor fraction of genes (390 genes, ∼1% of the total number of genes) had SV within their genic span including 2 kbp upstream region as putative promoter regions. These genes could be candidates for altered gene function between the accessions; some might be pseudogenized by frameshift-inducing indels. Our expression atlas indicated significantly lower expression levels for SV-harboring genes (Fig. 3B), with an even greater reduction observed when SVs overlapped coding sequences (CDS) (Figs. S6–S7). In a confirmatory leaf analysis based on alignment-derived counts using STAR, genes harboring SVs again exhibited larger absolute log_2_FCs than non-SV genes. Consistent with these overall trends, H4 *vs* YW differences were evident within leaf tissue (Fig. 3C and Fig. S4). Using a pseudo-alignment-based count matrix with the same model gave the same conclusion (Fig. S3), indicating the result is aligner-agnostic. SV-bearing genes did not show any particular tendency regarding TS expression (*τ* index; two-tailed Wilcoxon test, not significant). The geometric distribution of SVs on each chromosome does not show any common pattern across the genome, yet it is not uniform either (Fig. S8).

### Tissue-biased deployment of *FvTFL1* and validation by qPCR

**Figure 4:**
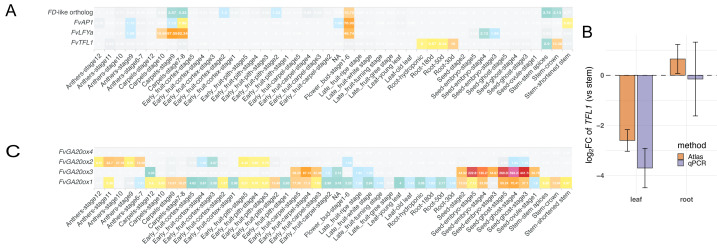
Heatmaps and a validation for one representative gene by qPCR. (A) Heatmap of tissue-biased expression generated with the Expression Comparator module of the Strawberry Atlas web interface. Rows (top to bottom): *FD*-like ortholog, *FvAP1*, *FvLFYa*, and *FvTFL1*; columns correspond to each tissue and stage profiled in the atlas. Color intensity indicates TPM values averaged across replicates. The heatmap highlights the biased expression of *FvTFL1*, *FvLFYa*, and *FvAP1* in stem apices and floral tissues with co-expression of the *FD*-like ortholog. (B) Bars show mean log_2_FC of *FvTFL1* relative to stem for the leaf/stem and root/stem contrasts; error bars indicate ± standard error across biological replicates. RNA-seq (TPM) values were computed per replicate on the log_2_(TPM + *ɛ*) scale (*ɛ* = 0.01 TPM) and summarized to obtain log_2_FC. qPCR fold change was derived on the ΔCt scale as log_2_FC =  − ΔΔCt. The horizontal dashed line marks zero (no change). Method differences within each contrast were assessed using a Wald test on the difference of estimates (no significant differences). (C) Heatmap of *GA20ox* family expression, generated as in panel A. Rows (top to bottom): *FvGA20ox4*, *FvGA20ox2, FvGA20ox3*, and *FvGA20ox1*. The heatmap highlights the seed-predominant signal of *GA20ox3*, the broad low-level expression of *GA20ox1*, and the absence of high *GA20ox4* expression in the surveyed tissues.

Having established robust tissue-level structure in the integrated atlas, we examined tissue-biased expression of the floral regulator *FvTFL1* as a representative validation. *FvTFL1* declines as *FvLFYa* and *FvAP1* increase during the apex transition, consistent with an *FD*-dependent antagonism at the apex (Lembinen et al., 2023). We asked whether this logic extends across tissues. In the atlas, leaf libraries were near background for *FvTFL1*, whereas stems including shoot apices showed low-to-moderate abundance (∼TPM 8; batch-corrected); root tips also showed moderate abundance (Fig. 4A). Our atlas and qPCR yielded concordant log_2_FC, confirming higher *FvTFL1* expression in shoot apices and root tips than in leaves (Fig. 4B). A putative *FD/FDP*-like bZIP ortholog (*FvesChr1G00292710*) was co-expressed in stems and in floral buds, in line with the canonical *FD*-dependent antagonism at the apex (Fig. 4A). By contrast, root tips displayed *FvTFL1* expression without detectable *FD/FDP*-like, *FvLFYa* or *FvAP1* signals in RNA-seq, a configuration not explained by the canonical apex model. Yet, the pattern is consistent with our qPCR validation (Fig. 4B).

### Web Interface for integrative analysis

To facilitate straightforward exploration of the gene expression atlas results, we developed and made publicly available a web-based database and browser using the recently established framework (Fernandez-Pozo & Bombarely, 2022). This interface allows users to easily examine the expression patterns of multiple genes of interest across various tissues. To demonstrate its practical utility, we highlight selected use cases that leverage key features of the interface.

#### Bridging v4 and v6 annotations *via* the web interface

Although the present study utilizes the v6 genome assembly, a substantial body of prior research has been established using the v4 annotation (Edger et al., 2018; Li et al., 2019). To facilitate the integration of these legacy data with current analyses, a reliable cross-version gene identifier (ID) map is indispensable. To our knowledge, no such systematic correspondence between the v4 and v6 gene models is publicly available. To address this, we established a correspondence lookup table between the genome versions. The mapping prioritizes syntenic evidence (Wang et al., 2012) and is further refined using orthologous group assignments and phylogenetic relationships inferred by OrthoFinder (Emms & Kelly, 2019). We have made this resource accessible through our web interface, which allows for intuitive bidirectional searches between v4 and v6 IDs. Leveraging the framework’s capabilities, the interface supports batch conversion of large gene lists, and the results can be downloaded in common formats such as CSV and Excel. This functionality provides a critical bridge, enabling researchers to seamlessly integrate the wealth of knowledge from previous v4-based gene expression studies with new findings derived from the v6 assembly. For bioinformaticians and users requiring greater detail, a comprehensive table that includes confidence levels and phylogenetic distances for each mapping is also available for download. This layered accessibility ensures that the resource can cater to the diverse needs of the research community.

#### Exploration of *GA20ox* family expression across tissues

As another use case of the expression atlas and the integrative capabilities of the web interface, we examined the TS expression patterns of the *GA20-oxidase* (*GA20ox*) gene family. This gene family plays a well-characterized role in gibberellin biosynthesis and has been implicated in axillary bud outgrowth and stolon formation in *F. vesca* (Mouhu et al., 2013; Tenreira et al., 2017). In particular, variation in *GA20ox4* has been implicated in the runnerless trait, as observed in YW (Joldersma et al., 2022) and other contexts (Tenreira et al., 2017). To evaluate the utility of the web interface and simultaneously assess the consistency of our dataset with prior reports, we visualized the expression levels of annotated *GA20ox* orthologs in the atlas. Notably, *GA20ox3* displayed sharp seed-predominant expression, and *GA20ox2* is expressed in early fruit tissues but absent in embryo as previously reported (Fig. 4C; Kang et al., 2013). *GA20ox4* expression was not detected at high levels in any of the surveyed tissues. However, quite low but consistent expression (TPM ∼ 0.3) was observed specifically in a few stem samples (Fig. 4C). It is possible that these samples contained axillary bud tissue, consistent with previous reports highlighting *GA20ox4* activity in axillary meristem (Tenreira et al., 2017). Interestingly, *GA20ox1* exhibited broad, nearly ubiquitous expression across all tissues except anthers, where *GA20ox2* is highly expressed. The corresponding web panel allows users to explore these expression patterns interactively, compare transcript abundance between tissues and stages, and map gene model differences across genome versions. The *GA20ox* family illustrates how the resource can be used to validate known phenotypic associations while also enabling discovery of TS regulatory patterns in a flexible, user-driven manner.

#### Transposable element-derived exonization captured in the expression atlas

**Figure 5:**
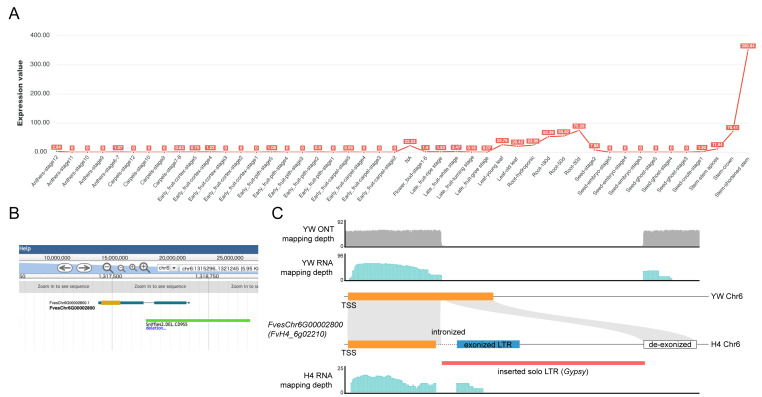
Characterization of *FvesChr6G00002800* (*FvH4_6g02210*) and 1.3-kbp insertion in H4 accession. (A) Expression level of the gene in the atlas, showing high expression level in stem, followed by root and leaf tissues. (B) Genome browser in the atlas showing the 1.3-kbp SV. (C) Schematic representation of *FvesChr6G00002800* and its counterpart in YW. This structural alteration is supported by ONT sequencing and RNA-seq mapping data in YW. A matching insertion was also identified in a recent haplotype-aware assembly of *F. vesca*, providing independent support for the variant.

As a final use case, we highlight a specific example in which a TE insertion results in the apparent exonization of an intronic region (Fig. 5). This event was identified through integration of SV calls and expression profiles visualized in the atlas. A ∼1.3-kbp insertion in the first intron and the second exon of *FvesChr6G00002800* (*FvH4_6g02210*) in the H4 accession was classified as a TE fragment based on RepeatMasker annotation. Superfamily assignments followed RepeatModeler2 and were corroborated by DeepTE predictions (Table S6). RNA-seq read coverage from H4 samples in this region revealed splicing consistent with the inclusion of a cryptic exon (Fig. 5C). Importantly, this SV was also observed in a recently assembled high-quality genome (GCA_964146915.1, GCA_964165485.1) from an independent UK-derived *F. vesca* line (Darwin Tree of Life Project Consortium, 2022), supporting the existence of the event (Fig. S9). This example demonstrates the utility of combining structural and expression data within the atlas to uncover transcriptionally active genomic alterations, including TE-derived exonization events that may otherwise escape annotation in reference genomes. Such cases further exemplify how the web interface can aid in discovering functionally significant genome variation beyond conventional gene models.

Taken together, these examples, including *GA20ox* family expression patterns and the TE-derived exonization event, demonstrate the ability of our web interface to integrate multiple genomic layers such as expression profiles, SVs, tissue specificity, and genome version mapping in a user-friendly environment. The interface is available at https://strawberryatlas.org/easy_gdb/. We are confident that these publicly available data and tools will prove to be a valuable resource for the strawberry research community.

### Usage notes

The atlas provides an interactive web portal for search, visualization, and download. Common use cases include:

• Converting gene IDs between v4 and v6 (*e.g.*, when only v4 IDs are available).
• Cross-tissue inspection of user-defined gene lists (*e.g.*, *TFL1*/*LFY*/*GA20ox*).
• Exporting TPM expression values for tissues of interest in multiple formats.
• Visual exploration of genes in their genomic context, including proximity to TEs or identified SVs.

A concise tutorial is available online, and a full usage guide is also provided in the Supplemental Information.

## Discussion

In this study, we constructed a comprehensive gene expression atlas for *F. vesca* by integrating hundreds of public RNA-seq datasets and supplementing them with newly generated data, ultimately covering 36,039 actively expressed genes (∼99% of the annotated set), which underscores both the richness of the *F. vesca* transcriptome and the value of combining publicly available datasets with a new dataset. A key outcome of this work is our identification of TS and HK genes across nine major tissue categories: for instance, photosynthesis-related genes in leaves and flavonoid-related genes in seeds and fruits highlight distinct metabolic requirements, while a set of the 719 constitutively expressed HK genes emphasizes the need to validate classic markers in a tissue- and genotype-specific manner. These TS and HK gene lists provide a valuable resource for functional studies and expression analyses in *F. vesca*.

Our atlas and qPCR data resolve two TS deployments of *FvTFL1* in *F. vesca* (Figs. 4A, 4B). At the floral tissues, *FvTFL1* decreases as *FvLFYa* and *FvAP1* rise and a putative *FD/FDP*-like factor is co-expressed, consistent with the canonical *FD*-dependent antagonism that buffers the floral transition (Lembinen et al., 2023). In root tips, *FvTFL1* accumulates while *FD/FDP*-like*, FvLFYa,* or *FvAP1* are not detected in either RNA-seq or qPCR (Figs. 4A, 4B), suggesting an *FD*-independent mode that may reflect partner switching to alternative partners outside floral development. This framework reconciles antagonism confined to the shoot apex with root-specific *FvTFL1* accumulation and preserves a gate for floral commitment in meristems. A dedicated follow-up will be required to identify the precise partners in root tips and to test *FD*-independence functionally.

The expression patterns observed among the *GA20ox* gene family members suggest possible subfunctionalization following gene duplication events (Force et al., 1999). Phylogenetic analysis by OrthoFinder indicates that *GA20ox2* (*FvesChr7G00336560; FvH4_7g12600*) and *GA20ox3* (*FvesChr7G00336570; FvH4_7g12610*) likely arose from a relatively recent tandem duplication event that occurred within a lineage of the Rosaceae, whereas *GA20ox1* (*FvesChr7G00355230; FvH4_7g28670*) and *GA20ox4* (*FvesChr2G00221480; FvH4_2g35050*) represent more ancient, divergent lineages (Fig. S10). Notably, *GA20ox3* exhibits a highly seed-specific expression pattern, and *GA20ox2* is primarily active in early fruit tissues (Kang et al., 2013), consistent with more specialized, stage- and tissue-specific roles. In contrast, *GA20ox1* is characterized by lower but widespread expression across a broad range of tissues and developmental stages, suggesting that it may retain a more basal function. This divergence in expression domains and intensities implies functional partitioning among family members. The patterns observed here are consistent with subfunctionalization models, in which duplicated genes partition ancestral functions or acquire novel regulatory inputs as discussed in another plant species (Sun et al., 2023).

The overall bias of SVs toward non-exonic regions indicates purifying selection on coding exons (Table S5), but the minority of exonic SVs identified may have outsized impacts on gene function or regulatory control. These observations align with data from other plant species, where insertion of TEs can either reduce expression *via* disruption of coding sequences or create novel isoforms through exonization (Kang et al., 2023). An example is the partial overlap of a TE with an exon region, yielding an alternative transcript structure in H4 compared to YW (Fig. 5). These observations suggest that TE insertions in *F. vesca* may still be a dynamic force in shaping gene architecture and expression patterns. *F. vesca* YW genome and distinct high-quality haplotype-aware assembly support that this could be a recent insertion in H4 lineage. OrthoFinder cluster illustrates that *FvesChr6G00002800* and paralogs are conserved in wild and cultivated strawberries but not conserved in other *Rosaceae* species we investigated. Nevertheless, our results suggest a potential contribution to intraspecific phenotypic variation, warranting further functional validation.

A key objective of this work is to provide the research community with a public, integrated resource, rather than a definitive functional dissection of specific genes or pathways. By merging large-scale expression data, SV analysis, and TE annotations into a user-friendly web platform, we enable investigators to rapidly query gene expression patterns, examine potential SV- or TE-related disruptions, and explore genotype-specific regulatory differences in *F. vesca*. Although the scope of conditions and tissue samples is broad, certain developmental stages and stress conditions remain underrepresented, and the current atlas does not fully resolve the impacts of subtle environmental differences among the datasets. Moreover, the functional significance of many SVs and TE insertions is yet to be experimentally validated. In future studies, controlled growth experiments or segregating populations could elucidate whether these variants have direct consequences for traits such as fruit quality, flowering, and runner formation.

Nevertheless, our integrated atlas provides a robust foundation for further hypothesis-driven research into *F. vesca* biology and its relevance to the cultivated strawberry, *F.* × *ananassa*, which partially descends from *F. vesca*-like subgenomes (Edger et al., 2019; Session & Rokhsar, 2023; Song et al., 2024). We should note that extending the present framework to the cultivated octoploid strawberry (*F.* × *ananassa*) is nontrivial. Given the relative abundance of public data for *F.* × *ananassa*, we assembled an analogous compendium and conducted a pilot analysis (Table S7). The results indicated diminished cross-project concordance (Fig. S11), consistent with reference bias arising from subgenome divergence, homeolog imbalance, and high standing variation. Notably, even among samples from the same tissue within the same project, pairwise correlations were consistently low (Fig. S11). In such contexts, single reference mapping could misassign reads, compress true expression differences, and hinder comparability across studies. Our observations suggest that the primary driver is not polyploidy itself but reference sample bias. When the genetic distance between the reference and the target cultivar is small, correlations approach the levels seen in *F. vesca* (Min et al., 2020; Guan et al., 2022; Knoch et al., 2025). While reference bias currently makes full integration nontrivial, we anticipate that technical limitations will be progressively alleviated, at which point integrating cultivated strawberry datasets should become feasible. As these technical limitations are addressed, we plan to extend the atlas to incorporate cultivated strawberry datasets. At the same time, these constraints would underscore the value of *F. vesca* as a tractable model for strawberry genomics.

Beyond reference-related heterogeneity, sequence-level variation provides a second axis of expression instability. In this context, recent work highlights gene-associated TE polymorphisms in *F. vesca* (Tossolini et al., 2025). Our tissue-resolved atlas offers the baseline needed to evaluate TE-expression coupling across tissues and stages. Given the relative simplicity of *F. vesca*, insights gained here may inform polyploid strawberry breeding, particularly in identifying candidate loci susceptible to SV- or TE-mediated regulation. We hope this publicly available dataset will accelerate gene discovery, facilitate cross-accession comparisons, and offer novel leads for unraveling the genetic basis of key horticultural traits.

## Conclusions

In conclusion, this study presents an integrative expression atlas of *F. vesca* constructed from a large collection of public and in-house RNA-seq datasets, curated and normalized to reveal robust tissue-level transcriptional landscapes. The resulting dataset enables fine-scale comparisons across developmental stages and accessions, facilitating insight into both constitutive and specialized gene expression. Notably, by leveraging this resource, we identified a previously underappreciated ubiquity in the expression of *GA20ox1*, contrasting with the more tissue-restricted patterns of other GA20ox family members. These findings exemplify the power of comprehensive atlases in uncovering nuanced transcriptional behaviors that may escape notice in narrower studies. By coupling expression atlases with genome structure annotations, we shed light on how SVs might shape the transcriptome of this diploid model. We anticipate that this resource-oriented approach, together with accessible web tools, will greatly enhance future functional genomics efforts in *Fragaria* and related crops.

## Supplemental Information

10.7717/peerj.20740/supp-1 Supplemental Information 1 Overview of the datasets The left and right panels show UMAP visualization of samples before and after the batch correction, respectively. Samples marked by green circle are H4 leaf samples sequenced in this study and dots show samples removed as outliers.

10.7717/peerj.20740/supp-2 Supplemental Information 2 Overview of the analyses and datasets derived from count-based metrics The left and right panels display UMAP embeddings of samples colored by (left) major tissue categories and (right) pre-merging categories, respectively (labels as in Figure S1). The top and bottom legends correspond to the left and right panels, respectively. Samples enclosed by green circles are H4 leaf samples sequenced in this study, and dots indicate samples excluded as outliers.

10.7717/peerj.20740/supp-3 Supplemental Information 3 Box plots showing the distribution of log_2_-transformed absolute fold-change in leaf tissue between genes associated or not associated with SVs Expression level was estimated by kallisto-based counts. Significance was determined using two-tailed Wilcoxon test. *P*-values are indicated by asterisks: *p* ¡ 0.001 (***), *p* ¡ 0.01 (**), and *p* ¡ 0.05 (*).

10.7717/peerj.20740/supp-4 Supplemental Information 4 Concordance of TPM- and count-based workflows assessed by hierarchical clustering The top panel reports the Adjusted Rand Index (ARI) after curation, whereas the bottom panel includes low-quality outlier samples.

10.7717/peerj.20740/supp-5 Supplemental Information 5 Heatmaps and box plots displaying the distribution of correlation coefficients for groups from the same tissue (organ) and/or BioProject (lab) The left panel illustrates the distributions before outlier removal and batch correction, while the right panel shows the distributions after these processing steps.

10.7717/peerj.20740/supp-6 Supplemental Information 6 Box plots showing the distribution of log_2_-transformed TPM in leaf tissue for genes harboring SVs within their genic spans, classified according to genomic regions We classified an SV as *SV-spanning* if it overlapped multiple gene features (e.g., both the coding sequence and an intron). Because many gene models do not consistently include 5′or 3′UTRs (non-CDS exons), we excluded SVs overlapping non-CDS exons from this classification to avoid potential biases in annotation. Significance was determined using two-tailed Wilcoxon test. Adjusted *P*-values were calculated using the BH method and are indicated by asterisks: *p*_adj_ ¡ 0.001 (***), *p*_*adj*_ < 0.01 (**), and *p*_*adj*_ < 0.05 (*).

10.7717/peerj.20740/supp-7 Supplemental Information 7 Box plots showing the distribution of log_2_-transformed absolute fold-change in leaf tissue for genes harboring SVs within their genic spans, classified according to genomic regions We classified an SV as *SV-spanning* if it overlapped multiple gene features (e.g., both the coding sequence and an intron). Because many gene models do not consistently include 5′or 3′UTRs (non-CDS exons), we excluded SVs overlapping non-CDS exons from this classification to avoid potential biases in annotation. Significance was determined using two-tailed Wilcoxon test. Adjusted *P*-values were calculated using the BH method and are indicated by asterisks: *p*_*adj*_ < 0.001 (***), *p*_*adj*_ < 0.01 (**), and *p*_*adj*_ < 0.05 (*).

10.7717/peerj.20740/supp-8 Supplemental Information 8 Genomic density plot of SVs on each chromosome. Green vertical lines represent centromeric positions

10.7717/peerj.20740/supp-9 Supplemental Information 9 The SV overlapping with * FvesChr6G00002800* (*FvH4_6g02210_*) is also observed in a distinct assembly released under Darwin Tree of Life project (GCA_964146915.1_, GCA_964165485.1_) The top track shows SV in Tier1, the second and the third track represent alignment between the assembly against the version 6 reference. The SV seems to exist as a homozygous variant in the sequenced sample.

10.7717/peerj.20740/supp-10 Supplemental Information 10 Gene tree of the GA20ox gene family inferred using OrthoFinder The displayed topology is derived from the species tree-aware inference implemented in OrthoFinder (resolved tree); therefore, traditional branch support values such as bootstrap scores are not applicable. Distinct colors indicate the major clades corresponding to the four GA20ox paralogs in *F. vesca*. The clade containing *GA20ox1, GA20ox2,* and *GA20ox3* appears to have diverged early from the *GA20ox4* lineage. Subsequent subfunctionalization within the *GA20ox1/2/3* clade likely occurred progressively during the evolution of eudicots.

10.7717/peerj.20740/supp-11 Supplemental Information 11 Heatmaps and box plots displaying the distribution of correlation coefficients for groups from the same tissue (organ) and/or BioProject (lab) for *Fragaria* x *ananassa* The left panel illustrates the distributions before outlier removal and batch correction, while the right panel shows the distributions after these processing steps.

10.7717/peerj.20740/supp-12 Supplemental Information 12 Supplemental tables

10.7717/peerj.20740/supp-13 Supplemental Information 13 Full Usage Guides

## Additional Information and Declarations

### DNA Deposition

The following information was supplied regarding the deposition of DNA sequences: The raw sequencing reads are available at NCBI SRA: PRJNA1265872. Every external RNA-seq dataset re-analysed here was downloaded from the SRA and can be retrieved with the accession numbers listed in Table S1.

### Data Availability

The following information was supplied regarding data availability: All processed data, together with the interactive browser for the expression atlas and SV/TE tracks, are available at: https://strawberryatlas.org/easy_gdb. The same application can be deployed locally via a Docker. The code is available at GitHub and Zenodo: - https://github.com/yfukasawa/strawberryatlas_template. - Yoshinori Fukasawa. (2025). yfukasawa/strawberryatlas_template: v0.1 for public release (0.1). Zenodo. https://doi.org/10.5281/zenodo.17685719
The code used for gene mapping is available at Github and Zenodo: - https://github.com/yfukasawa/gene_version_mapper
- Yoshinori Fukasawa. (2025). yfukasawa/gene_version_mapper: v1.0 for public release (v1.0). Zenodo. https://doi.org/10.5281/zenodo.17685727. The processed TPM matrices and structural variants are available at Figshare: - Odagiri, Minto; Thagun, Chonprakun; Kurokura, Takeshi; Suzuki, Tomohiro; Kodama, Yutaka; Fukasawa, Yoshinori (2025). Gene expression data of Fragaria vesca. figshare. Dataset. https://doi.org/10.6084/m9.figshare.29125859.v1
- Odagiri, Minto; Thagun, Chonprakun; Kurokura, Takeshi; Suzuki, Tomohiro; Kodama, Yutaka; Fukasawa, Yoshinori (2025). Structural variants of Fragaria vesca. figshare. Dataset. https://doi.org/10.6084/m9.figshare.29125805.v2.
