# Supplementary figures and images for: Strawberry atlas: Fragaria vesca gene expression atlas for strawberry genomics
Source: PeerJ. 2026 Feb 5;14:e20740. doi: 10.7717/peerj.20740 (PMC12883160; doi:10.7717/peerj.20740)

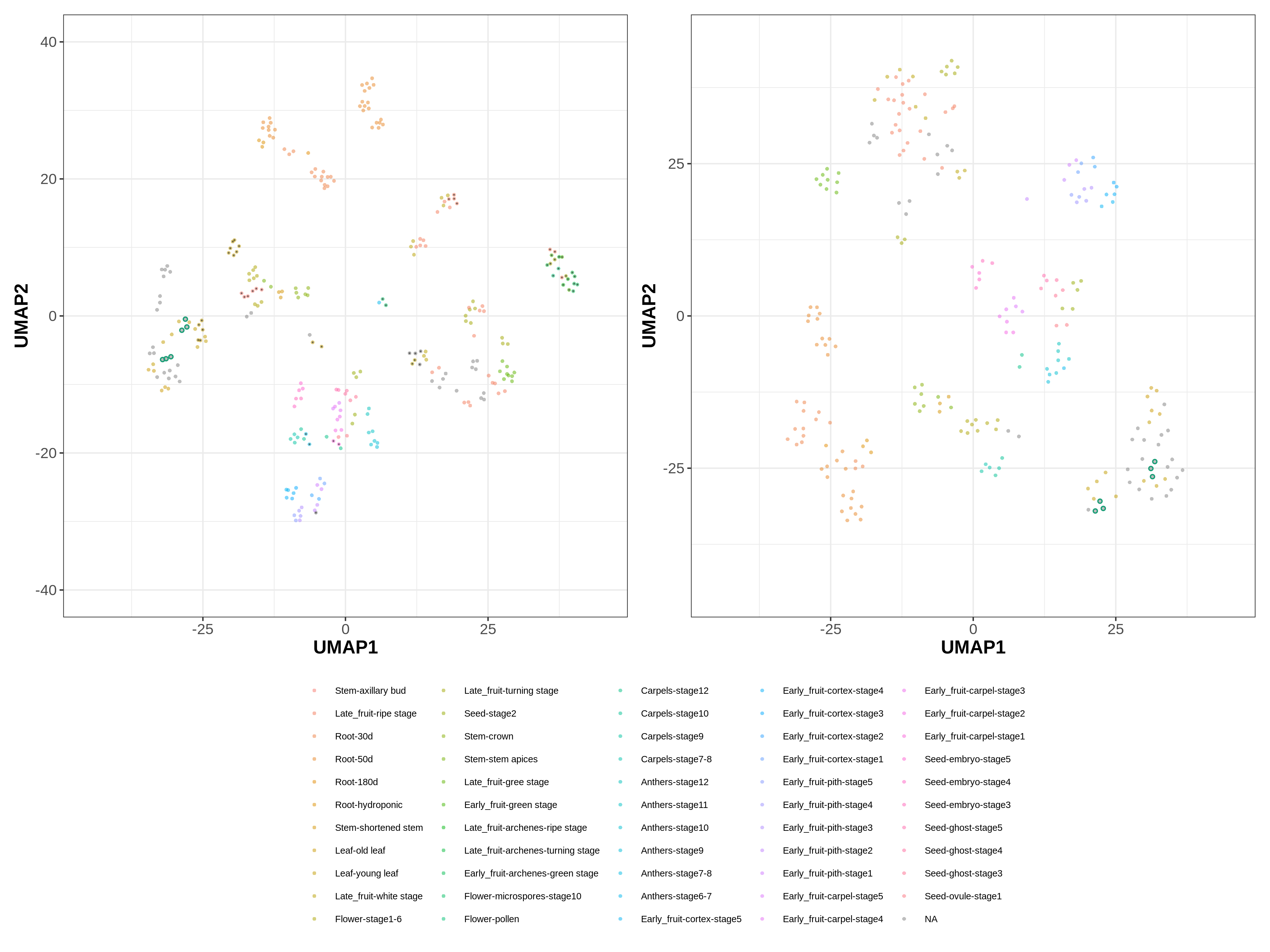

Supplement: Supplemental Information 1 — The left and right panels show UMAP visualization of samples before and after the batch correction, respectively. Samples marked by green circle are H4 leaf samples sequenced in this study and dots show samples removed as outliers. [file peerj-14-20740-s001.png]

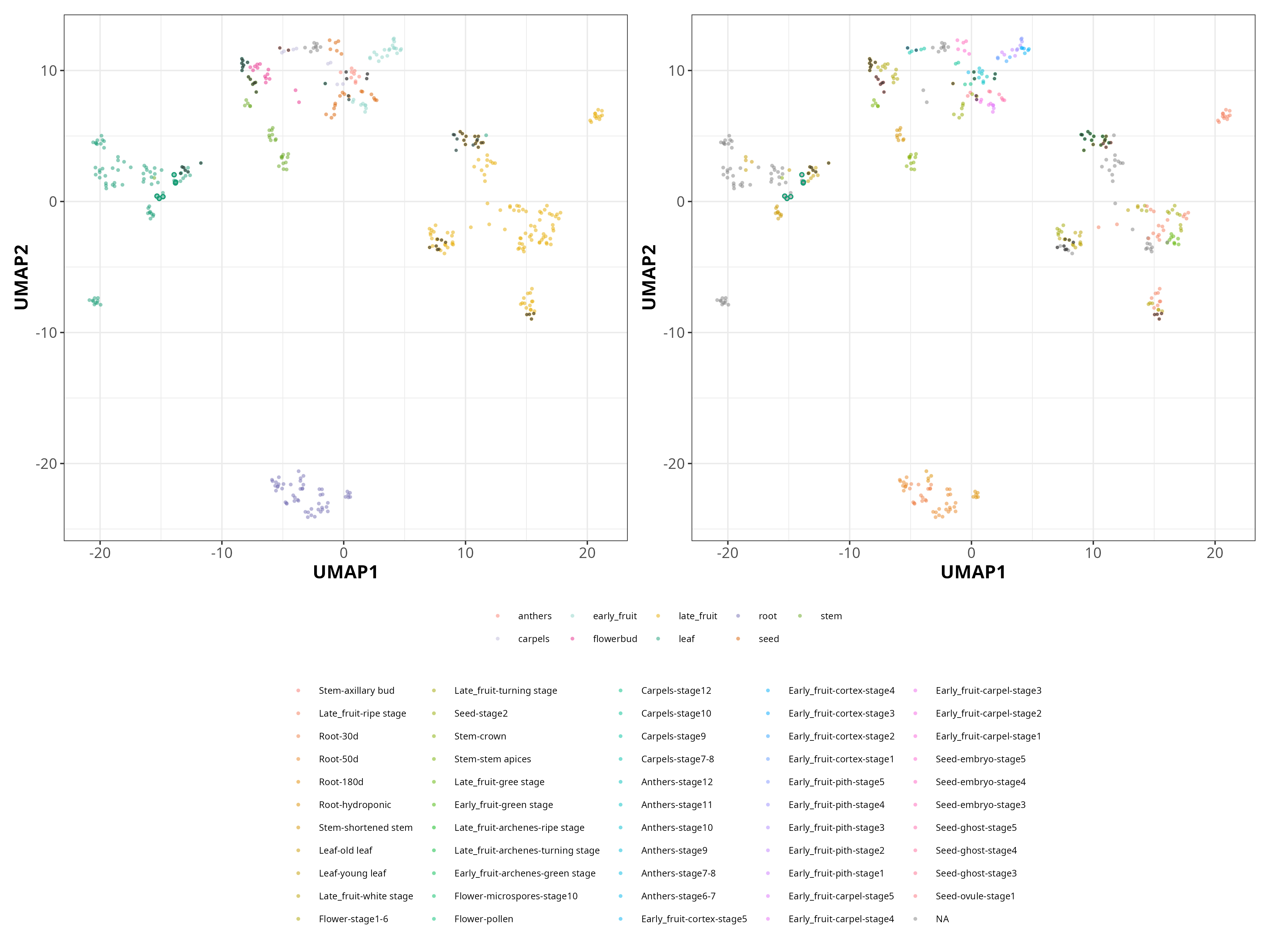

Supplement: Supplemental Information 2 — The left and right panels display UMAP embeddings of samples colored by (left) major tissue categories and (right) pre-merging categories, respectively (labels as in Figure S1). The top and bottom legends correspond to the left and right panels, respectively. Samples enclosed by green circles are H4 leaf samples sequenced in this study, and dots indicate samples excluded as outliers. [file peerj-14-20740-s002.png]

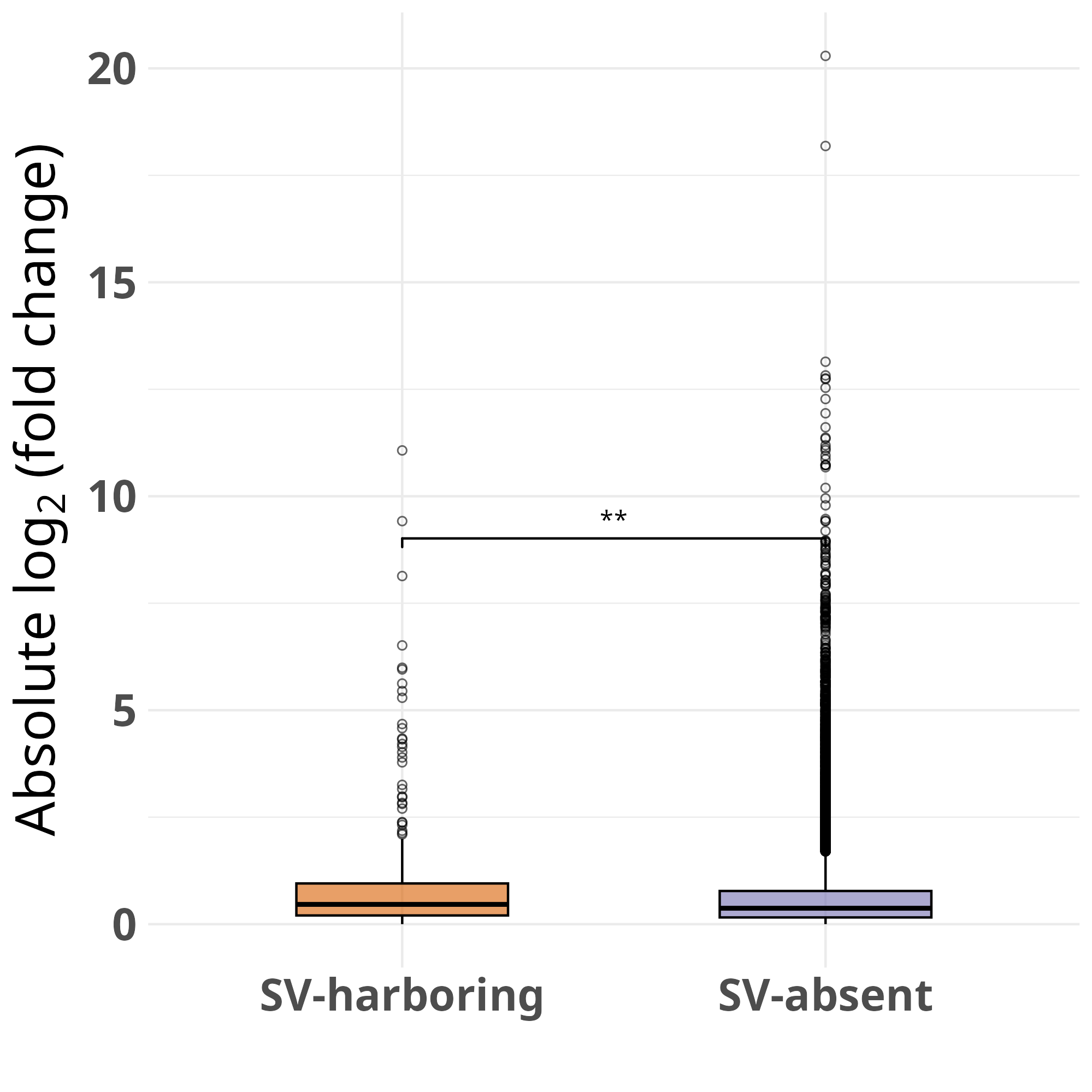

Supplement: Supplemental Information 3 — Expression level was estimated by kallisto-based counts. Significance was determined using two-tailed Wilcoxon test. P-values are indicated by asterisks: p ¡ 0.001 (***), p ¡ 0.01 (**), and p ¡ 0.05 (*). [file peerj-14-20740-s003.png]

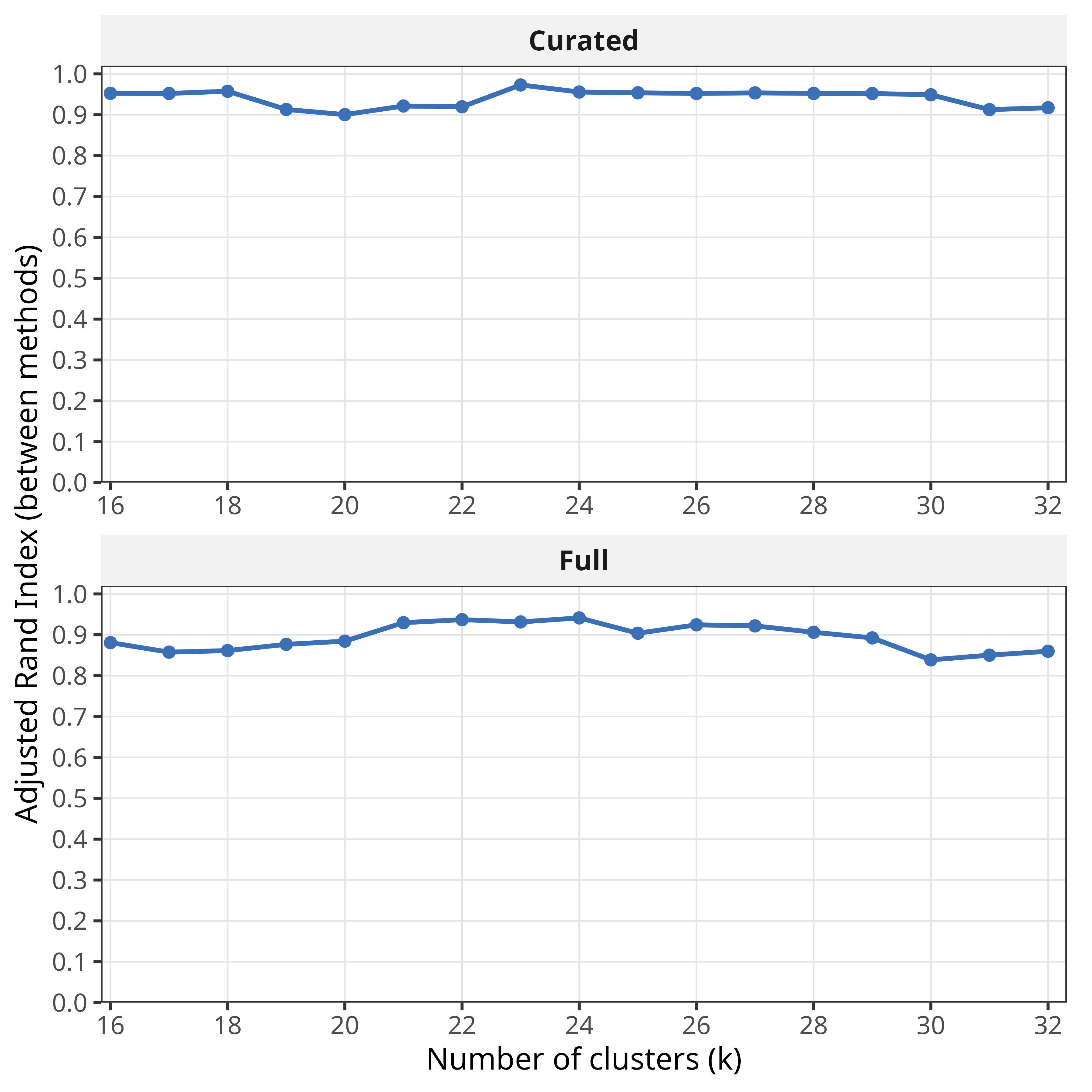

Supplement: Supplemental Information 4 — The top panel reports the Adjusted Rand Index (ARI) after curation, whereas the bottom panel includes low-quality outlier samples. [file peerj-14-20740-s004.png]

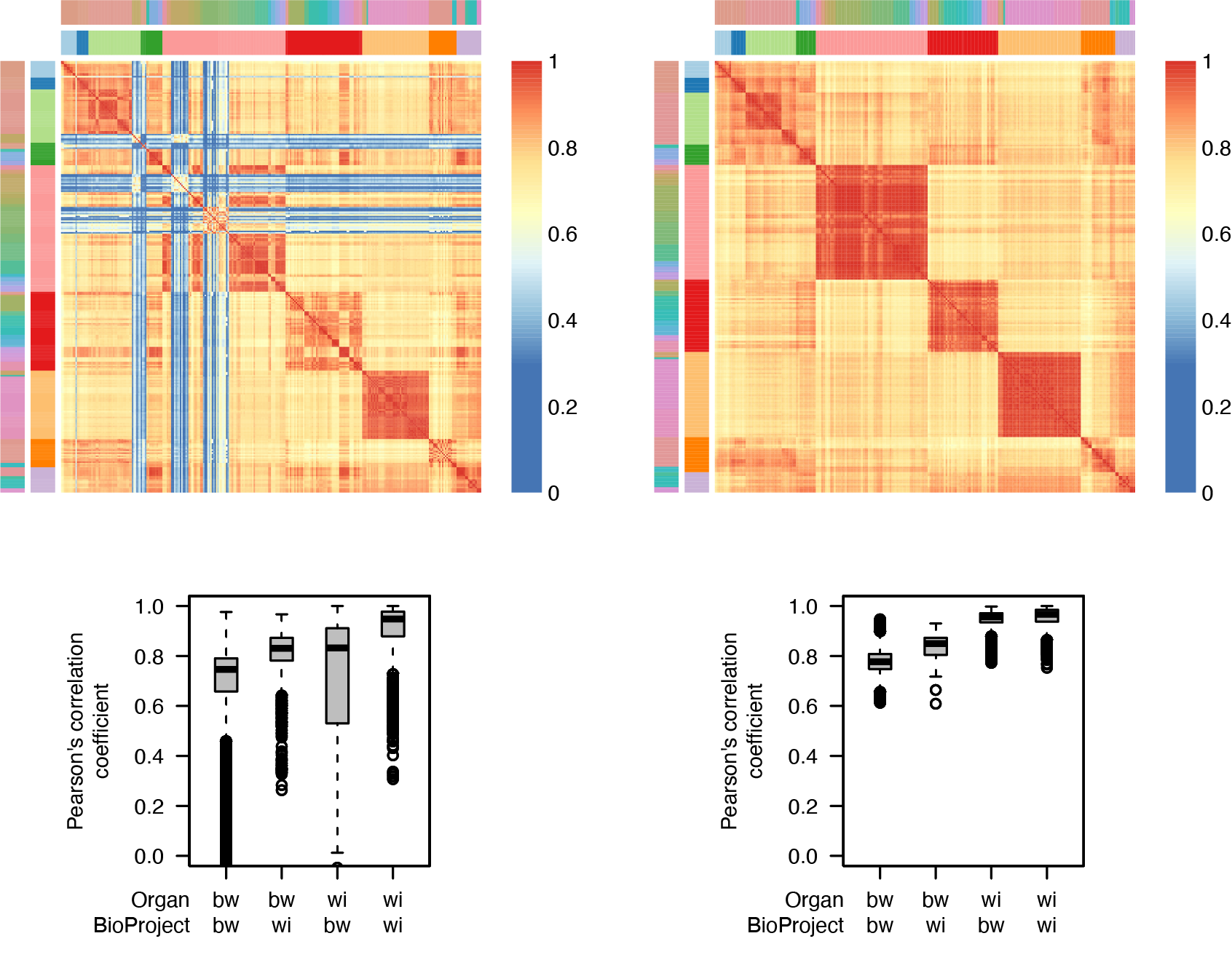

Supplement: Supplemental Information 5 — The left panel illustrates the distributions before outlier removal and batch correction, while the right panel shows the distributions after these processing steps. [file peerj-14-20740-s005.png]

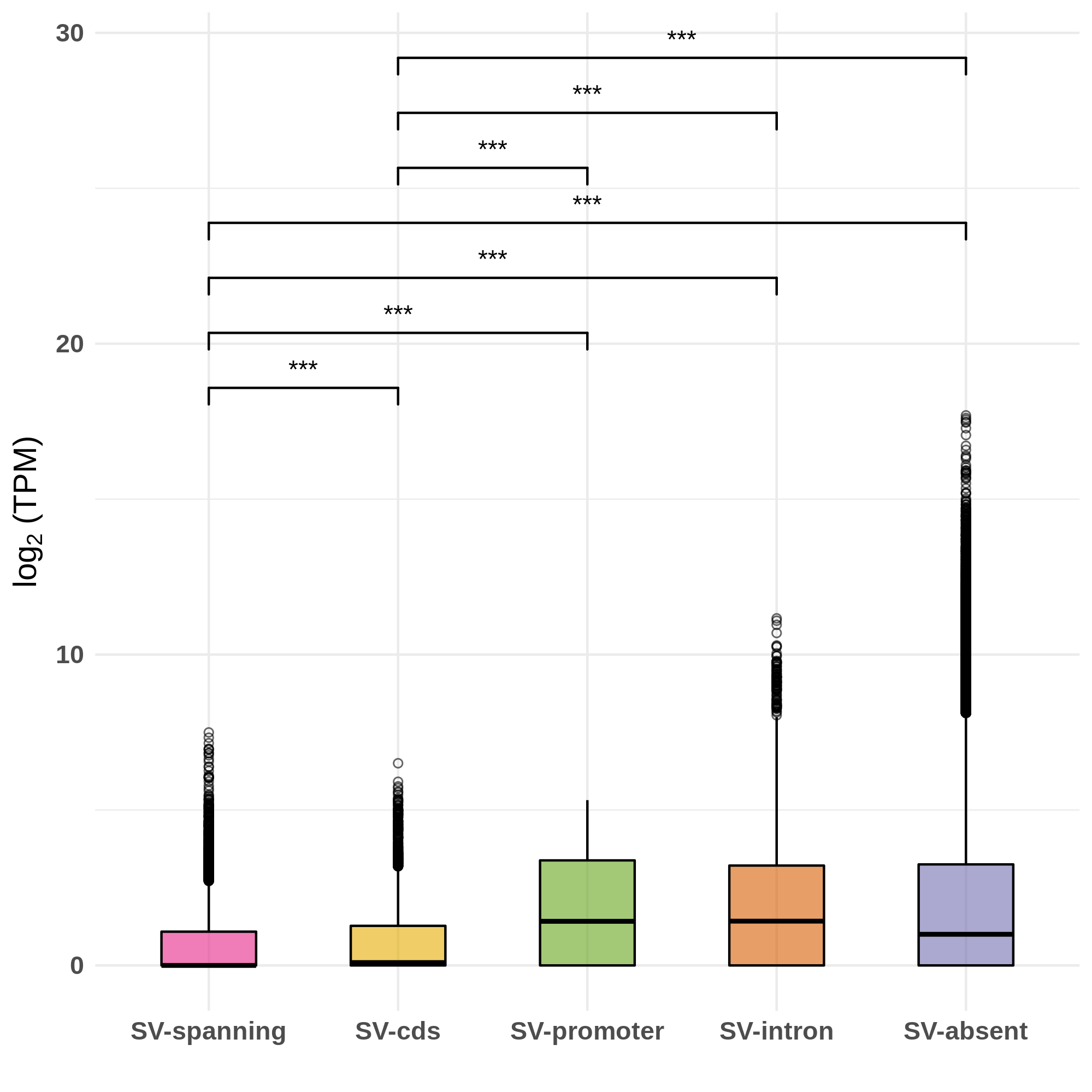

Supplement: Supplemental Information 6 — We classified an SV as SV-spanning if it overlapped multiple gene features (e.g., both the coding sequence and an intron). Because many gene models do not consistently include 5′or 3′UTRs (non-CDS exons), we excluded SVs overlapping non-CDS exons from this classification to avoid potential biases in annotation. Significance was determined using two-tailed Wilcoxon test. Adjusted P-values were calculated using the BH method and are indicated by asterisks: p adj ¡ 0.001 (***), padj < 0.01 (**), and padj < 0.05 (*). [file peerj-14-20740-s006.png]

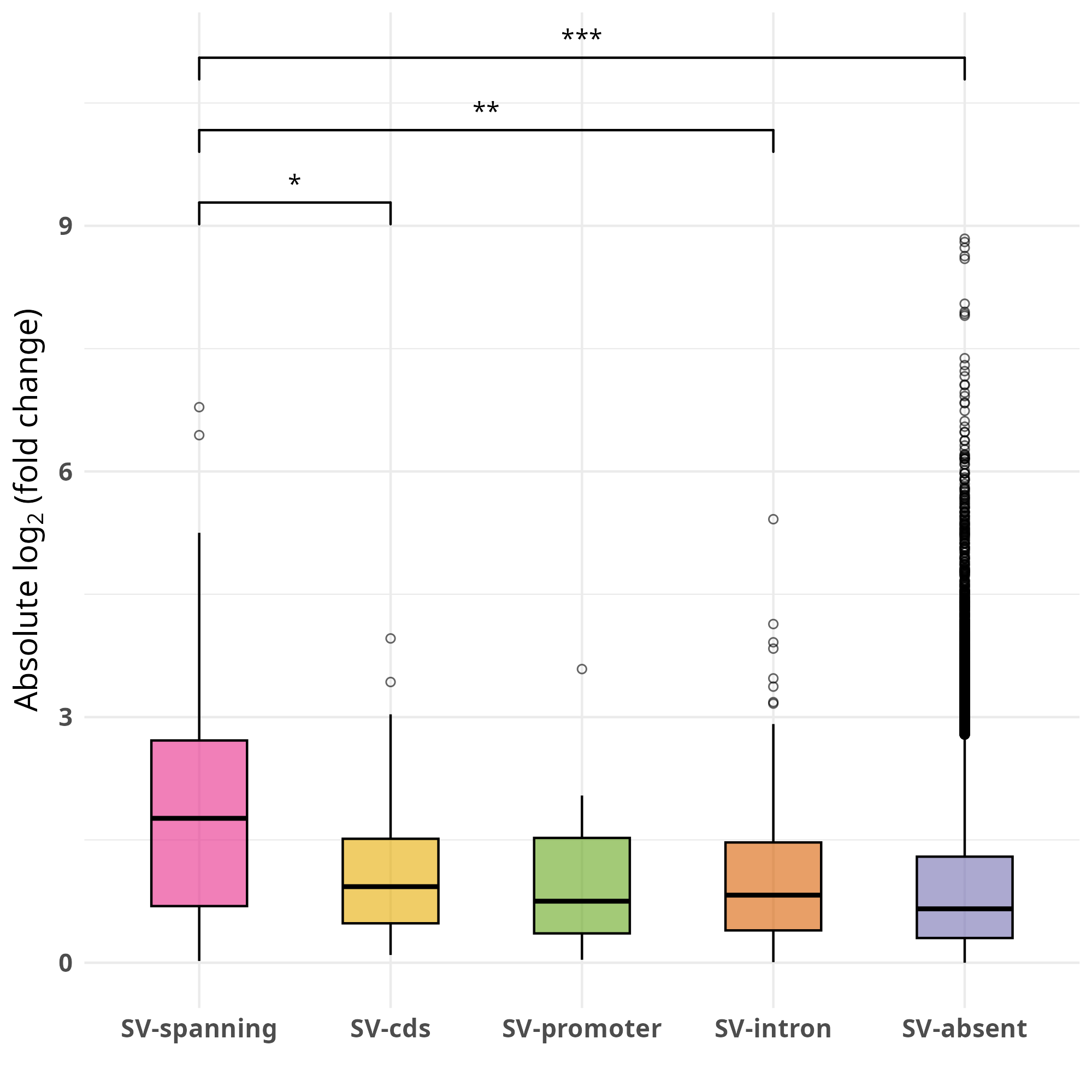

Supplement: Supplemental Information 7 — We classified an SV as SV-spanning if it overlapped multiple gene features (e.g., both the coding sequence and an intron). Because many gene models do not consistently include 5′or 3′UTRs (non-CDS exons), we excluded SVs overlapping non-CDS exons from this classification to avoid potential biases in annotation. Significance was determined using two-tailed Wilcoxon test. Adjusted P-values were calculated using the BH method and are indicated by asterisks: padj < 0.001 (***), padj < 0.01 (**), and padj < 0.05 (*). [file peerj-14-20740-s007.png]

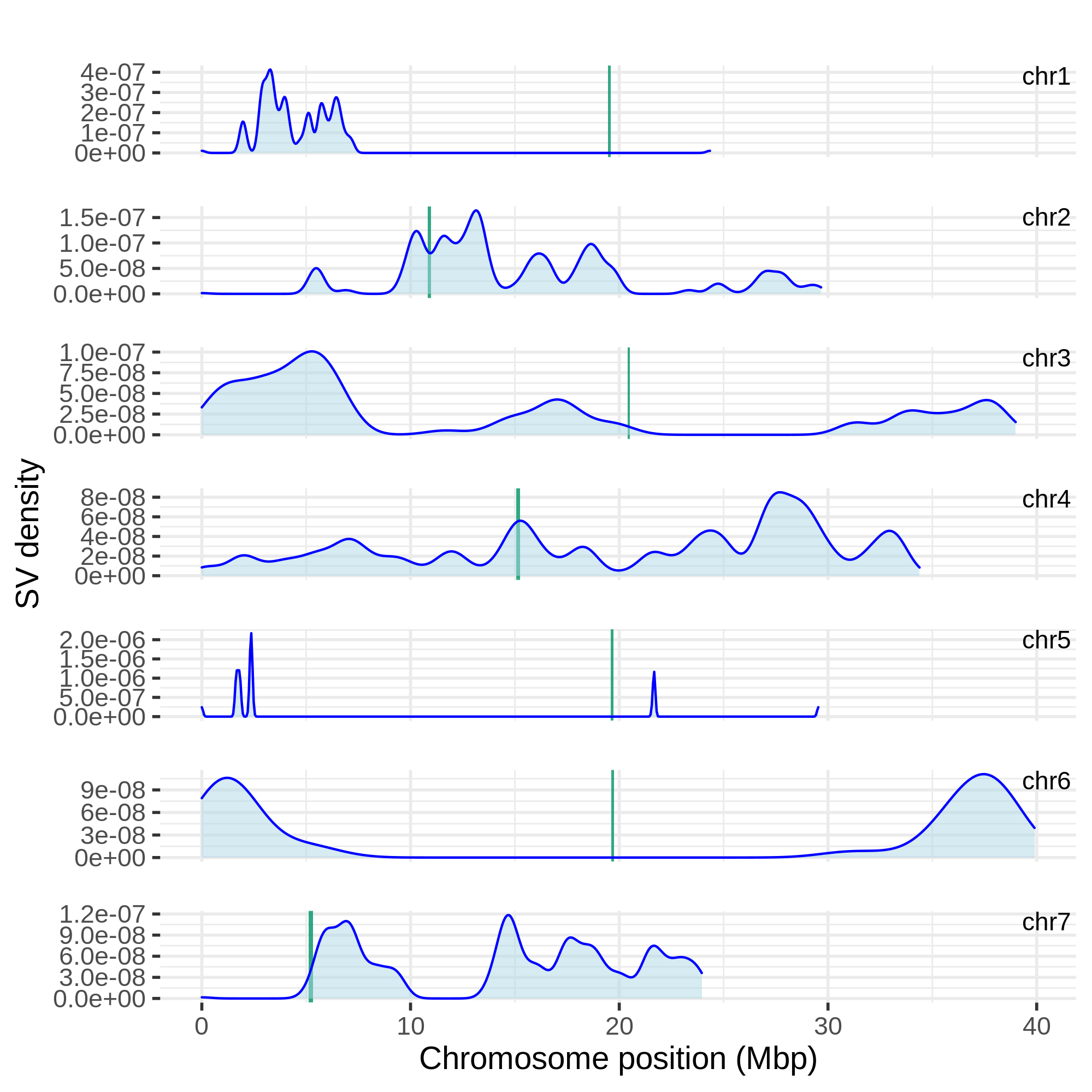

Supplement: Supplemental Information 8 [file peerj-14-20740-s008.png]

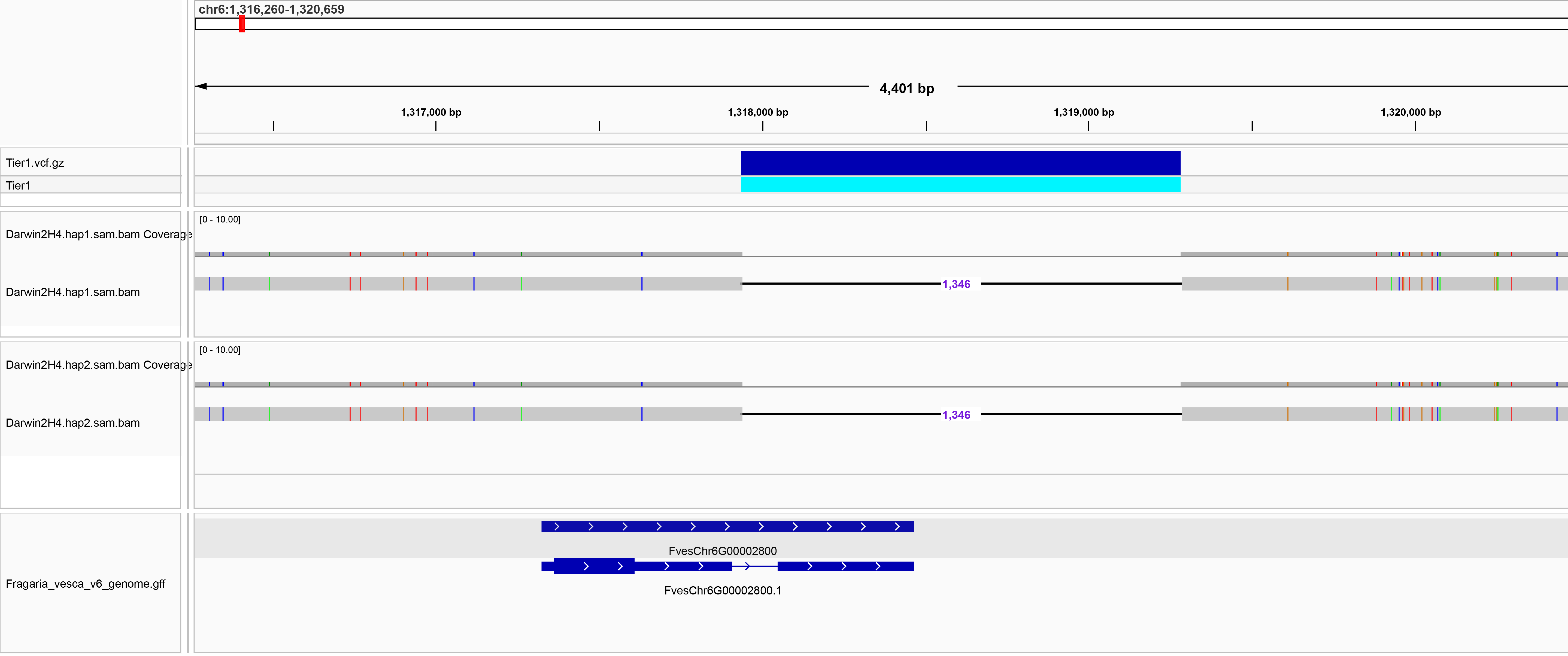

Supplement: Supplemental Information 9 — The top track shows SV in Tier1, the second and the third track represent alignment between the assembly against the version 6 reference. The SV seems to exist as a homozygous variant in the sequenced sample. [file peerj-14-20740-s009.png]

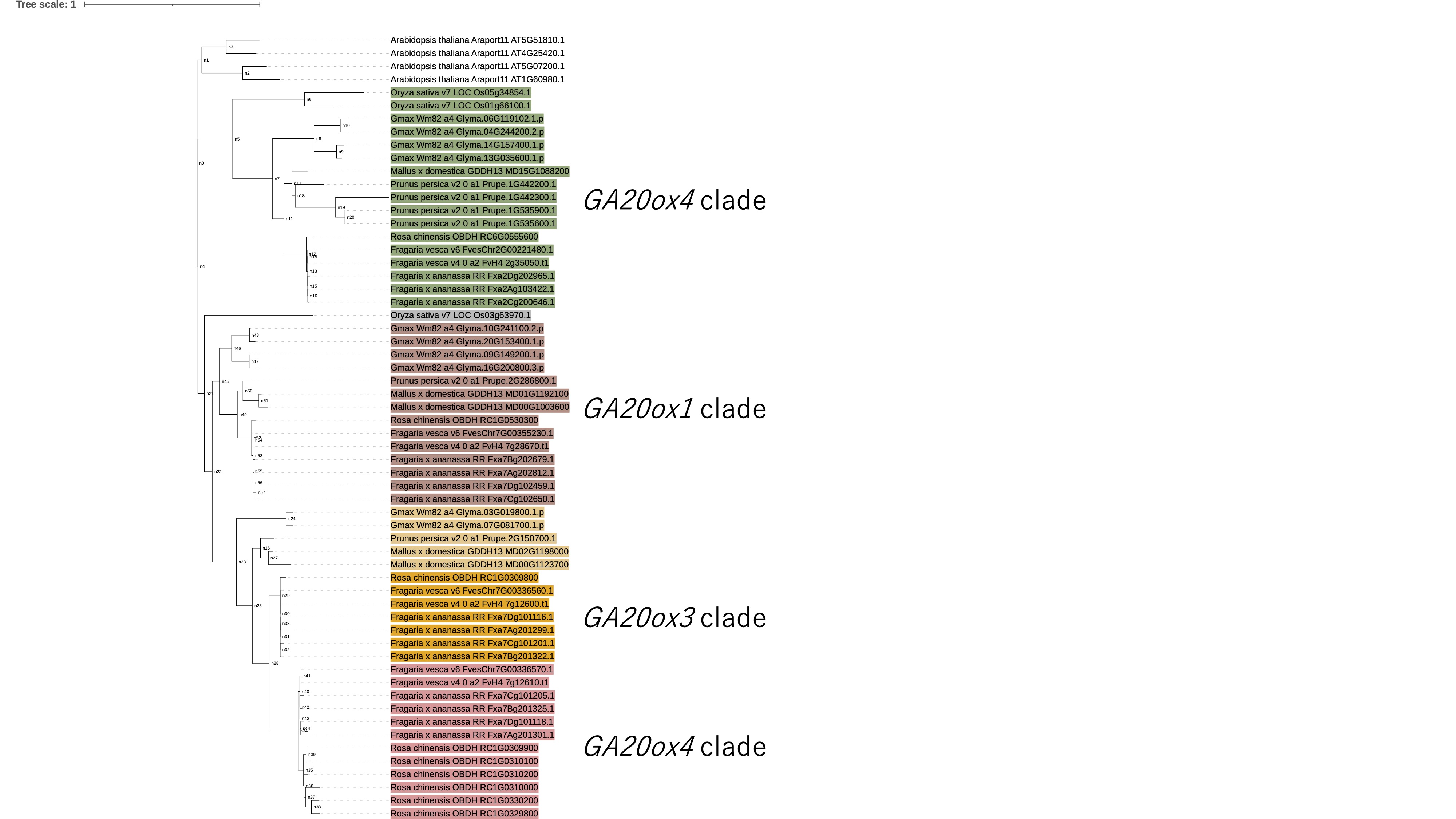

Supplement: Supplemental Information 10 — The displayed topology is derived from the species tree-aware inference implemented in OrthoFinder (resolved tree); therefore, traditional branch support values such as bootstrap scores are not applicable. Distinct colors indicate the major clades corresponding to the four GA20ox paralogs in F. vesca. The clade containing GA20ox1, GA20ox2, and GA20ox3 appears to have diverged early from the GA20ox4 lineage. Subsequent subfunctionalization within the GA20ox1/2/3 clade likely occurred progressively during the evolution of eudicots. [file peerj-14-20740-s010.jpg]

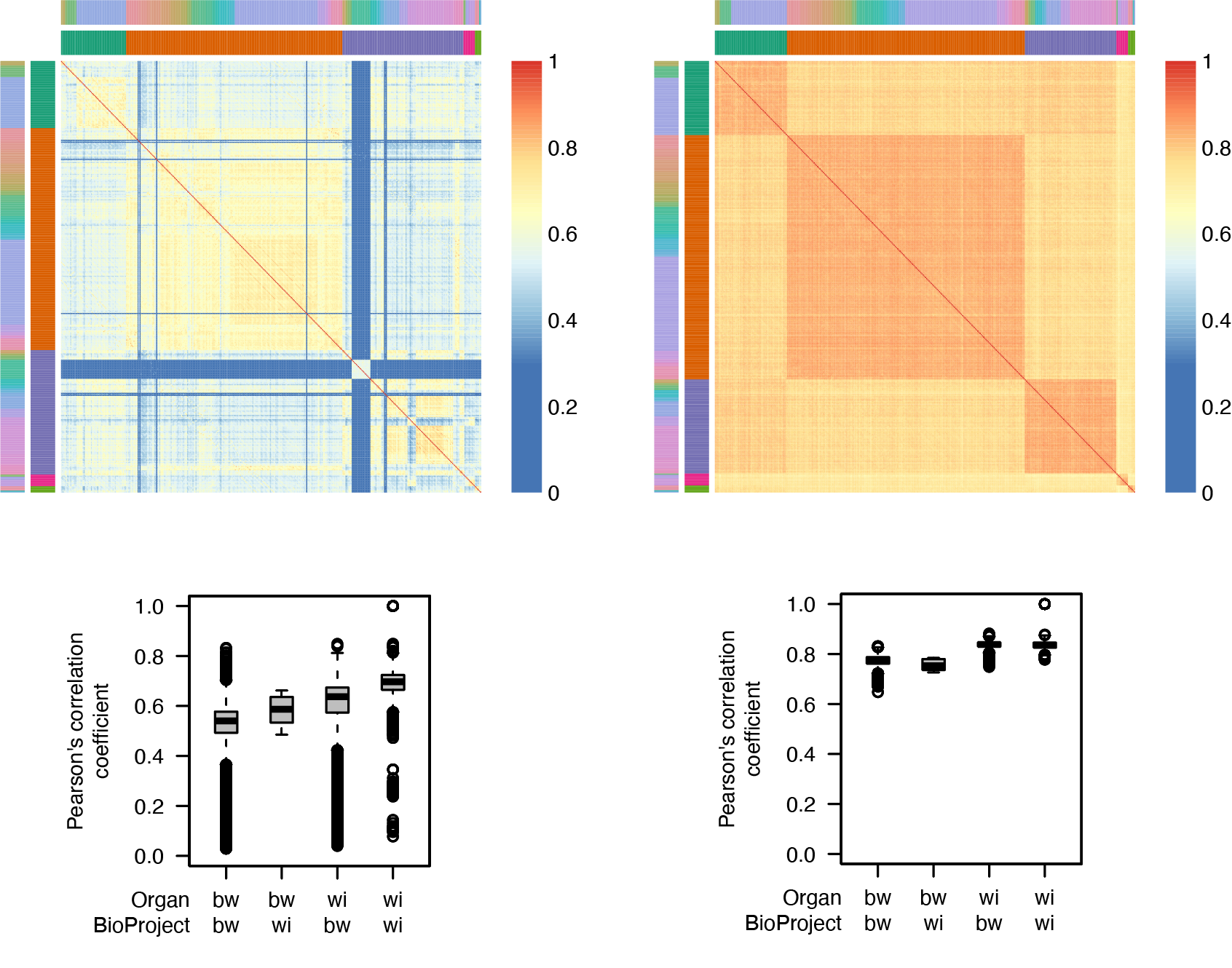

Supplement: Supplemental Information 11 — The left panel illustrates the distributions before outlier removal and batch correction, while the right panel shows the distributions after these processing steps. [file peerj-14-20740-s011.png]
