# Supplementary material for: Strawberry atlas: Fragaria vesca gene expression atlas for strawberry genomics
Source: PeerJ. 2026 Feb 5;14:e20740. doi: 10.7717/peerj.20740 (PMC12883160; doi:10.7717/peerj.20740)
Supplement: Supplemental Information 13 [file peerj-14-20740-s013.pdf]

# Full Usage Guide

## Overview

This atlas provides a harmonized, cross-study expression resource for *Fragaria vesca* across major tissues and stages. The web portal supports interactive search, visualization, and data download, and includes utilities for v4↔v6 gene-ID conversion and genomic-context views (TE/SV proximity).

- Reference & IDs: gene models in v6 (T2T) and legacy v4 (FvH4); both are supported.
- Expression units: TPM matrices for exploration; raw counts are available for downstream analyses.
- Batch handling: See Methods for SVA/normalization details; exported TPMs match the matrices underlying the figures unless otherwise noted.

---

## 1. Converting gene IDs between v4 and v6

### Purpose

Integrate legacy analyses (v4) with new v6-based work, or move between resources that use different gene models.

### How to

1. Open Tools → Gene Version Lookup.

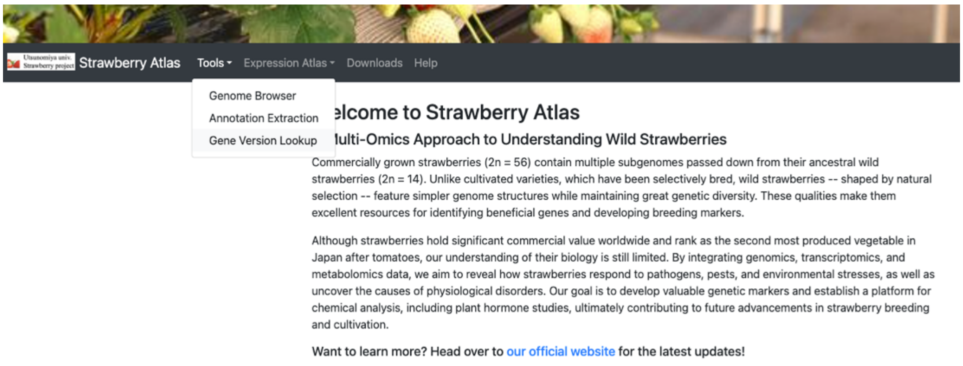


2. Paste or upload a list of gene IDs.

    - Input formats: one ID per line.
3. Choose conversion direction (v4→v6, v6→v4).
4. Click search → preview the mapping.

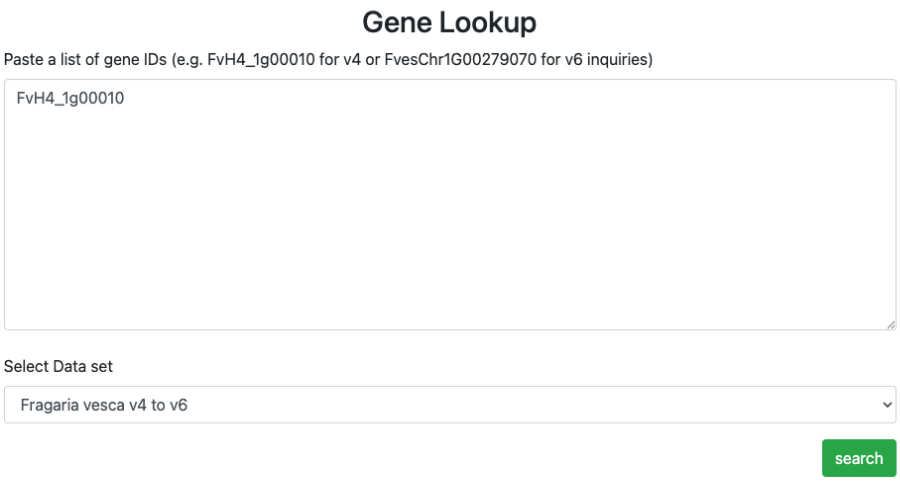


5. Download as CSV/Excel; the file includes input_id and matched_id.

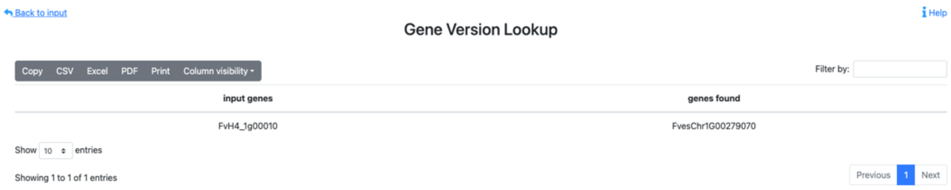


### Notes & caveats

- Some genes split/merge across versions.
- If you see no_hit, confirm the ID formatting (e.g., FvH4_1g00010 for v4 or FvesChr1G00279070 for v6).

---

## 2. Expression level inspection of user-defined gene lists

### Purpose

Check the expression level of genes of interest (e.g., *TFL1/LFY/GA20ox*) in the specified tissue group.

## How to

1. Go to Expression atlas → Expression viewer.

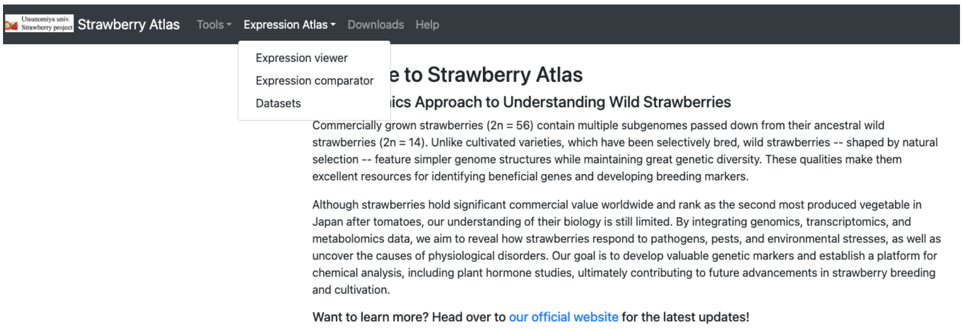


2. Provide a list of IDs in **v6**. Mixed versions are NOT acceptable.

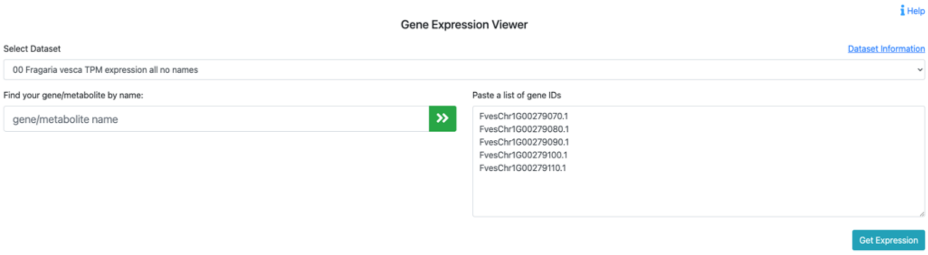


3. Select Dataset. Datasets having 00 prefix are groups merged into major tissue groups (regardless of stages and tissues with small number samples are merged). If you would like to focus on more on specific tissue/stage, select datasets having 01 prefix (e.g. 01 Fragaria vesca TPM expression anthers).

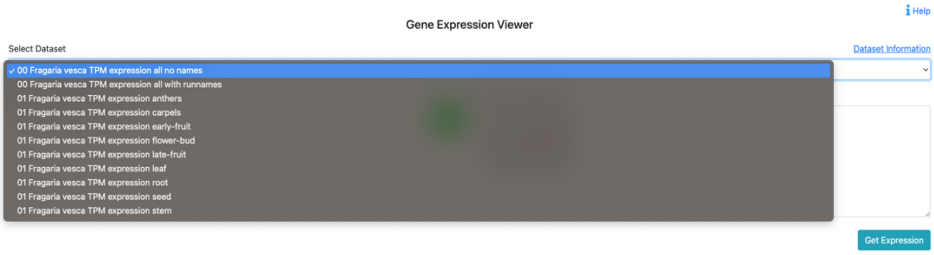


4. Click "Get Expression" button.
5. Click visualization type:

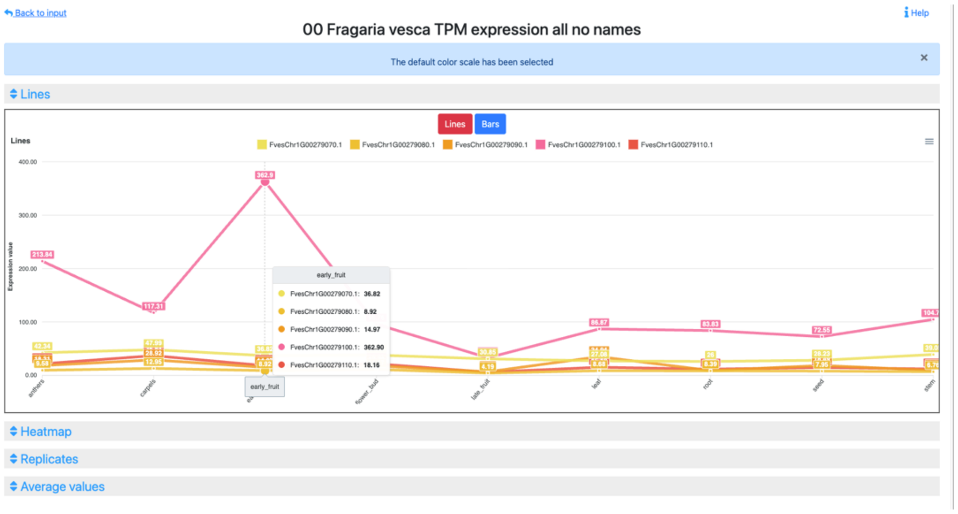


- Tissues × genes heatmap (TPM, log-scaled).
- Line plots per tissue.

6. Export figures (PNG/SVG) or underlying values (CSV/TSV).

### Interpretation tips

- TPM values are comparable within gene across samples. Between-gene comparisons require caution due to length/GC effects.

---

## 3. Cross-tissue inspection of user-defined gene lists

### Purpose

Check the expression level of genes of interest (e.g., *TFL1/LFY/GA20ox*) across the selected tissue groups. Here, expression levels of the genes can be compared across selected datasets (tissue/stages) at once.

### How to

1. Go to Expression atlas → Expression comparator.

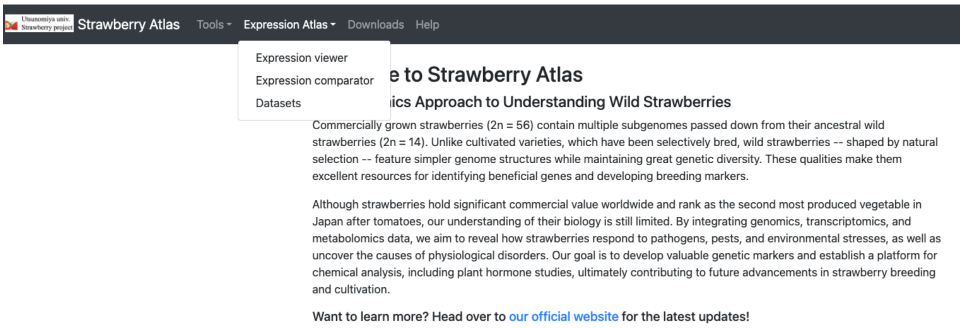


2. Provide a list of IDs in **v6**. Mixed versions are NOT acceptable.
3. Select multiple datasets. Datasets having 00 prefix are group into major tissue groups (regardless of stages and tissues with small number samples are merged). If you would like to focus on specific tissue/stages, select datasets having 01 prefix (e.g. 01 Fragaria vesca TPM expression anthers AND 01 Fragaria vesca TPM expression carpels).

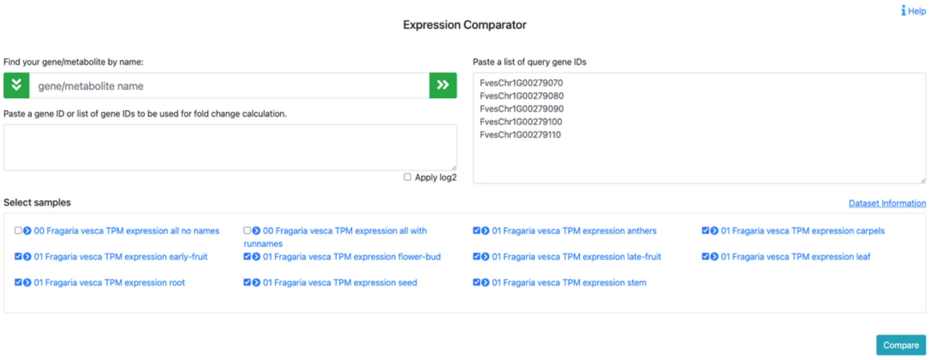


4. Click “Compare” button.
5. Click visualization type:

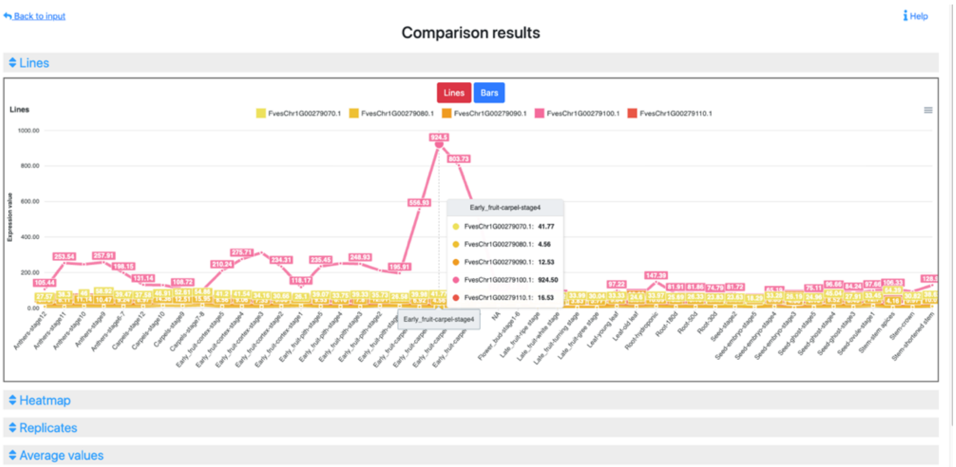

- Tissues × genes heatmap (TPM, log-scaled).
- Line plots per tissue.

6. Export figures (PNG/SVG) or underlying values (CSV/TSV).
   - When exporting figures for plots, click hamburger menu button on top right. SVG, PNG, or CSV.
   - When exporting tables for average values, click the format of the file to be downloaded.

### Interpretation tips

TPM values are comparable within gene across samples. Between-gene comparisons require caution due to length/GC effects.

---

## 4. Download expression matrices and metadata

### Purpose

Bring atlas data into your local computer.

### How to

1. Open Download.

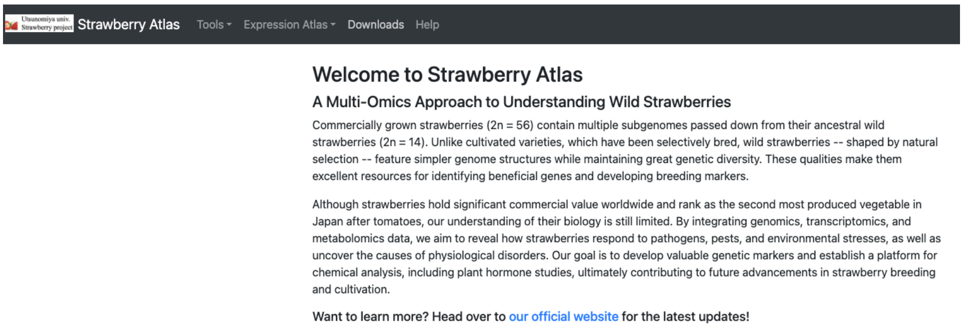


2. Choose data type and file.
   - Expression datasets:
     1. TPM (batch-corrected, each tissue type).
   - Genome annotation:
     1. TE annotation

2. ID mapping file
- Structural variants:
    1. Tier1/2/3
3. Simply click the name of the file to download !

**Download Files**

**Expression datasets**

| File | Last modified | Size |
|---|---|---|
| Fragaria vesca TPM expression all with runnames.tsv | 2025-04-21 | 98.7 MB |
| Fragaria vesca TPM expression all.tsv | 2025-04-21 | 98.7 MB |
| Fragaria vesca TPM expression anthers.tsv | 2025-04-21 | 4.8 MB |
| Fragaria vesca TPM expression carpels.tsv | 2025-04-21 | 4.1 MB |
| Fragaria vesca TPM expression early-fruit.tsv | 2025-04-21 | 11.8 MB |
| Fragaria vesca TPM expression flower.tsv | 2025-04-21 | 5.5 MB |
| Fragaria vesca TPM expression late-fruit.tsv | 2025-04-21 | 27.7 MB |
| Fragaria vesca TPM expression leaf.tsv | 2025-04-21 | 16.7 MB |
| Fragaria vesca TPM expression root.tsv | 2025-04-21 | 19.7 MB |
| Fragaria vesca TPM expression seed.tsv | 2025-04-21 | 8.5 MB |
| Fragaria vesca TPM expression stem.tsv | 2025-04-21 | 5.4 MB |
| Fragaria vesca log2-FC leaf H4 vs YW.csv | 2025-04-23 | 3.2 MB |

**Genome annotation**

| File | Last modified | Size |
|---|---|---|
| Fragaria_vesca_v6_genome.fasta.out.gff | 2024-12-20 | 27.7 MB |
| v6_v4_all_mapping_with_confidence.csv | 2025-07-03 | 2.9 MB |

**Structural variants**

| File | Last modified | Size |
|---|---|---|
| Tier1.vcf.gz | 2025-04-23 | 217.8 KB |
| Tier1.vcf.gz.tbi | 2025-04-23 | 6.4 KB |
| Tier2.vcf.gz | 2025-04-23 | 241.7 KB |
| Tier2.vcf.gz.tbi | 2025-04-23 | 2.9 KB |
| Tier3.vcf.gz | 2025-04-23 | 1.3 MB |
| Tier3.vcf.gz.tbi | 2025-04-23 | 23.4 KB |

---

## 5. Visual genomic context (TE/SV proximity)

### Purpose

Assess whether a gene sits near transposable elements (TEs) or a known structural variant (SV).

### How to

1. Open Tools → Genome Browser.

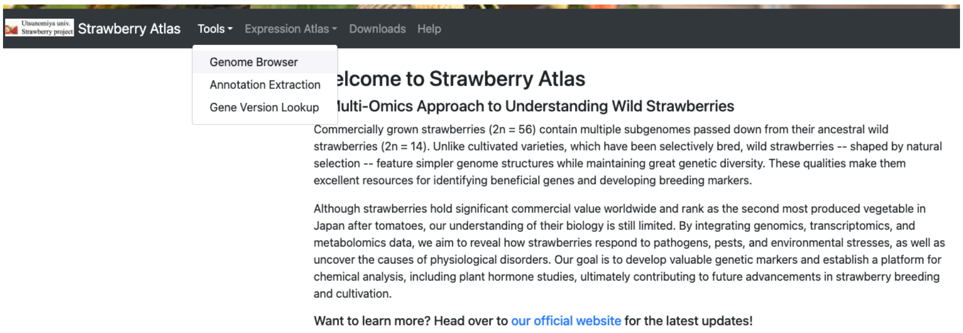


2. The viewer shows gene models, nearby TEs, and SV calls (tiers 1–2 high-confidence).

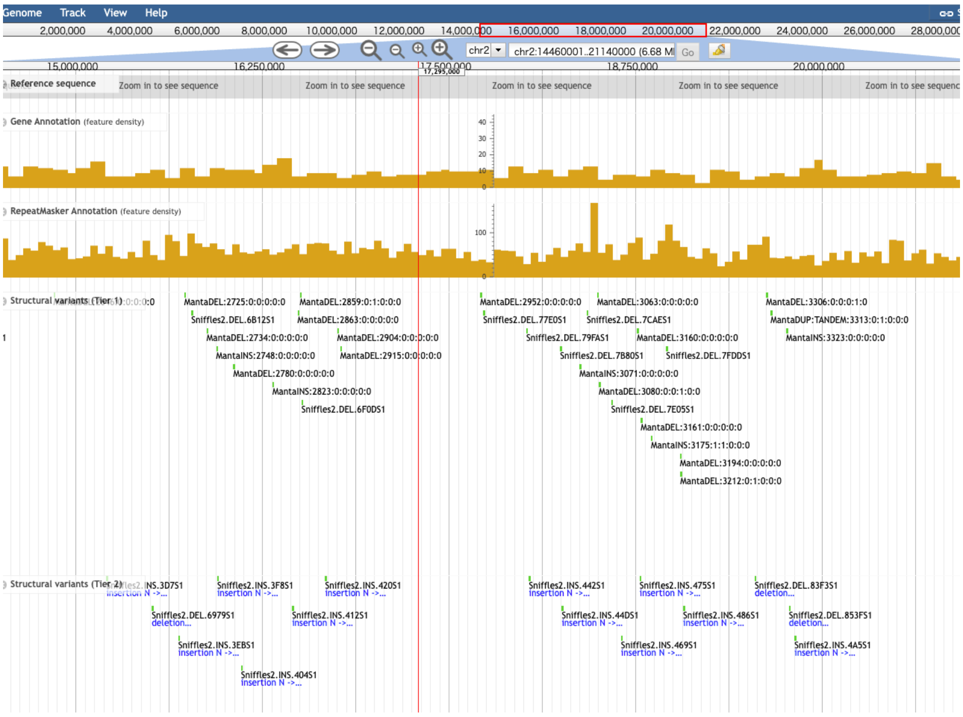


3. Hover to see distance (bp) and annotations.
4. Export snapshot (PNG/SVG) for figures or BED for intervals.

## Notes

- TE categories follow RepeatModeler2 classifications; Unknown indicates insufficient evidence.
- SVs are H4↔YW comparisons.

---

## 6. Known caveats & best practices

- **Between-gene comparisons** of TPM are not strictly quantitative. For hypothesis tests, use raw counts with appropriate normalization.
- **Low-abundance genes** near the detection limit can be unstable. Consider minimum TPM thresholds (e.g., TPM ≥ 1 for inclusion in certain metrics) as described in Methods.
- **Cross-version mapping** (v4↔v6): split/merged statuses require manual review when biological conclusions hinge on exact gene identity.
- **Batch effects**: the portal displays batch-corrected summaries; for custom analyses, consult metadata and re-fit your model as needed.
- **TE/SV context**: proximity does not imply causality. Use as a prioritization signal; follow-up analyses (e.g., allele-aware mapping, methylation) are recommended for mechanistic claims.

---

## 8. Troubleshooting (FAQ)

**Q: My v4 ID does not convert to v6.**
A: First, check the prefix (FvH4_), possible typos, and whether the gene was retired or merged between versions. If your query used the high-confidence (HC) gene set, try switching to the full dataset—some IDs exist only outside the HC subset and will not convert under HC-only mode. If the converter still returns no_hit, the gene may be version-specific (present only in v4 or only in v6). In that case, use sequence-based search (e.g., BLAST) to locate the corresponding locus. When using sequence-based search, be aware of the risk of inadvertently matching paralogs.

**Q: TPM looks different from a previous study.**
A: Library composition and normalization differ across studies. Our TPMs are generated under a uniform pipeline and corrected using SVA.

---

## 9. Contact & support

- **Web Help**: *https://strawberryatlas.org/easy_gdb/help/00_help.php*

- **Support**: the corresponding author
- **Issue reporting**: *https://github.com/yfukasawa/strawberryatlas_template*

---
